# It is not just the category: behavioral effects of fMRI-guided electrical microstimulation result from a complex interplay of factors

**DOI:** 10.1093/texcom/tgac010

**Published:** 2022-02-26

**Authors:** Satwant Kumar, Eline Mergan, Rufin Vogels

**Affiliations:** Laboratorium voor Neuro- en Psychofysiologie, Department of Neurosciences, KU Leuven, Herestraat 49, 3000 Leuven, Belgium; Leuven Brain Institute, KU Leuven, Herestraat 49, 3000 Leuven, Belgium; Center for Perceptual Systems, University of Texas at Austin, Austin, TX 78712, USA; Laboratorium voor Neuro- en Psychofysiologie, Department of Neurosciences, KU Leuven, Herestraat 49, 3000 Leuven, Belgium; Leuven Brain Institute, KU Leuven, Herestraat 49, 3000 Leuven, Belgium; Laboratorium voor Neuro- en Psychofysiologie, Department of Neurosciences, KU Leuven, Herestraat 49, 3000 Leuven, Belgium; Leuven Brain Institute, KU Leuven, Herestraat 49, 3000 Leuven, Belgium

**Keywords:** body patch, electrical microstimulation, inferior temporal cortex, macaque, visual categorization

## Abstract

Functional imaging and electrophysiological studies in primates revealed the existence of patches selective for visual categories in the inferior temporal cortex. Understanding the contribution of these patches to perception requires causal techniques that assess the effect of neural activity manipulations on perception. We used electrical microstimulation (EM) to determine the role of body patch activity in visual categorization in macaques. We tested the hypothesis that EM in a body patch would affect the categorization of bodies versus objects but not of other visual categories. We employed low-current EM of an anterior body patch (ASB) in the superior temporal sulcus, which was defined by functional magnetic resonance imaging and verified with electrophysiological recordings in each session. EM of ASB affected body categorization, but the EM effects were more complex than the expected increase of body-related choices: EM affected the categorization of both body and inanimate images and showed interaction with the choice target location, but its effect was location-specific (tested in 1 subject) on a millimeter scale. Our findings suggest that the behavioral effects of EM in a category-selective patch are not merely a manifestation of the category selectivity of the underlying neuronal population but reflect a complex interplay of multiple factors.

## Introduction

Causal techniques in which neural activity is manipulated are necessary to address the contribution of a brain area to behavior (Jazayeri and Afraz 2017). One causal technique that manipulates neural activity is intracranial electrical microstimulation (EM), where stimulation currents excite a cluster of neurons. In visual discrimination tasks (e.g. in middle temporal area [MT], Salzman et al. 1992, and in inferior temporal [IT] cortex, Verhoef et al. 2012), low current EM has been shown to change the choice of monkeys predictably. EM has also been used to investigate the contribution of IT neuronal clusters that show selectivity for the category of faces (Afraz et al. 2006). In 1 study (Afraz et al. 2006), EM was used to excite macaque IT neuronal clusters that showed a higher average response to faces compared to objects, which increased the frequency of face choices in a faces versus objects categorization task.

Functional magnetic resonance imaging (fMRI) studies have identified areas in human and monkey occipito-temporal cortex that are selectively activated by bodies relative to objects (Downing et al. 2001; Tsao et al. 2003; Popivanov et al. 2012). Subsequent studies have shown that single units’ spiking activity and high gamma band local field potential (LFP) power in the fMRI-defined body patches of the macaque superior temporal sulcus (STS) is on average greater to images of bodies compared to images of other categories such as of faces and objects (Popivanov et al. 2014; Kumar et al. 2017; Bao et al. 2020).

Lesion and, in particular, transcranial magnetic stimulation (TMS) studies in humans provide some evidence that the body category-selective extrastriate body area contributes causally to body perception (reviewed in Downing and Peelen 2016). However, for the more ventral and anterior fusiform body area (FBA), no TMS studies exist due to technical constraints. The present study aimed to examine the causal role of the macaque’s anterior STS body patch (ASB; Kumar et al. 2017), a possible FBA homolog (Caspari et al. 2014). As we showed before that ASB neurons had sufficient information to decode bodies versus objects (Kumar and Vogels 2019), we studied the effect of ASB EM on body categorization. We tested the hypothesis that EM in ASB would affect specifically the categorization of bodies versus objects, but not, or much less so, of other visual categories (e.g. faces vs. objects; Schalk et al. 2017). Following a previous study (Afraz et al. 2006), we added visual noise to the images, increasing the categorization difficulty and allowing measurements of psychometric functions with and without EM. We used 4-legged mammals as a “body” category as ASB responds strongly to these stimuli (Kumar et al. 2017; Bao et al. 2020). Furthermore, macaque monkeys can categorize animals versus objects (Fabre-Thorpe et al. 1998).

We found that EM affected not only body versus object categorization but also face versus object and house versus object choices. Manipulation of the behavioral task and visual field location of the choice targets showed that the behavioral effect of ASB EM was modulated by the task context and the target hemifield location. The results revealed that not only the stimulus category but a complex interplay of multiple factors also determine the impact of EM of a category-selective region on choices in a categorization task.

## Materials and methods

### Subjects

Two male rhesus monkeys (*Macaca mulatta*, weight: 6–9 kg), MB and MG, participated in this study. Both monkeys were implanted with a magnetic resonance compatible headpost. Also, recording chambers targeting the fMRI-defined patches in the STS were implanted. The surgery details have been previously described (Popivanov et al. 2014). The National and European laws regarding animal care and experimental procedures were followed and the study was approved by the Animal Ethics Committee of the KU Leuven (protocol P229/2014). All surgeries were performed under isoflurane anesthesia, and every effort was made to minimize discomfort.

### Stimuli

Two sets of visual stimuli were used in the study. The first used a subset of that of Popivanov and coworkers (Popivanov et al. 2014). This set will be referred to as the “search stimulus set” and it contains images of both inanimate and animate categories. The second stimulus set was created de novo for the behavioral experiments and consisted of different categories—animals, faces, houses, and objects. This set will be referred to as the “categorization stimulus set.”

#### Search stimulus set

The stimuli have been previously described (Popivanov et al. 2012), and only a brief description is provided here. Six classes of achromatic images, 4-legged mammals, human and monkey bodies (excluding the head), human faces, and human-made objects (matched either to the human or the monkey bodies aspect ratio) served as stimuli. Each class contained 10 different images. The headless human and monkey bodies were shown in different postures and varied in viewpoint (profile to frontal views).

#### Categorization stimulus sets

**Fig. 1:**
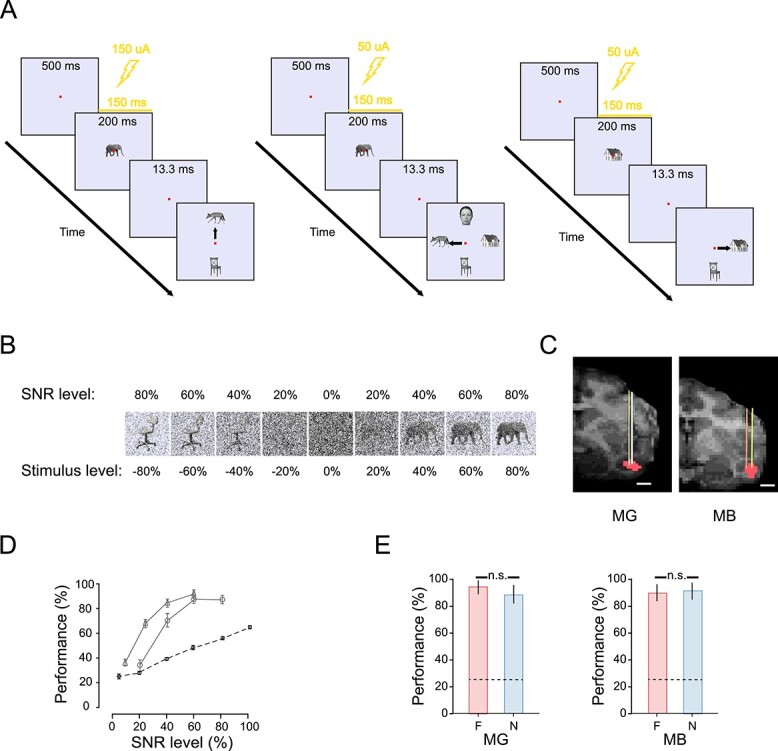
Tasks, anterior STS body patch (ASB), and controls. A) Categorization tasks. Left: 2CC task with fixed saccade target locations. The tasks included categorization of animals versus objects, faces versus objects, and houses versus objects. The animals versus objects task is shown as an example. A red fixation dot appeared for 500 ms after which the cue stimulus was presented with or without EM (150 μA). After an ISI of 13.33 ms, the monkey was required to saccade (black arrow) to the correct image target indicating the category of the cue. Middle: 4CC task. Similar to the 2CC task, a cue appeared following the presentation of only the red fixation dot. The cue could be an image from 4 categories: animals, faces, houses, and objects. After an ISI of 13.33 ms, the monkey had to make a saccade to 1 of the 4 target images that matched the category of the cue. The targets on the horizontal axis were switched between hemifields for the 2 monkeys. Right: 2CC categorization tasks with different target locations. The task structure was identical to the 2CC described above except that the spatial locations of the targets for the category choices were identical to those of the 4CC task. In these 2CC tasks, monkeys had to categorize animals versus objects, houses versus objects (shown in the figure), faces versus houses, faces versus objects, faces versus animals, and houses versus animals. In 50% of the trials of all the tasks, EM (lightning bolt icon) of 150-ms duration with a delay of 50 ms was administered during the presentation of the cue. In all tasks, correct choices were followed by a fluid reward. B) Examples of stimuli with different SNRs and stimulus levels. The images were overlaid with noise (“salt-and-pepper”) by replacing a fixed percentage of image pixels with an equal number of black and white noise pixels. An SNR level of i.e. 20% corresponds to 80% of the image being masked by noise. The stimulus level is a signed SNR, with positive values corresponding to images of animals and negative values images of nonanimals in this example. C) Anterior STS body patch (ASB, red) on a coronal MRI section, which was defined using a threshold of *t* = 7.0. Colored lines indicate estimated electrode locations targeting ASB. The scale bar corresponds to 5 mm. D) Performance of monkeys in trials without EM (solid gray lines) and a linear decoder trained on AlexNet conv1 layer activations (black stippled line) for the 4CC task as a function of SNR. Error bars correspond to standard errors of the mean. Triangles and circles correspond to the categorization performance of MG (*n* = 6,460 trials) and MB (*n* = 1,263 trials), respectively. E) Performance in the 4CC task with novel and familiar stimuli compared; 60% familiar and 40% novel stimuli of each category (animal, face, house, and object) were presented with an SNR of 80% and 60% for MB and MG, respectively. The percentage of correct choices for the first presentation of each stimulus is plotted for the familiar (red bar) and novel (blue bar) stimuli for MB and MG separately. Error bars indicate 95% CIs, which were computed using the binomial distribution approximation. The chance level of 25% is indicated by the dotted horizontal lines. Abbreviation: n.s. : not significant.

We created sets of animals and objects, faces and objects, houses and objects, animals and faces, faces and houses, animals and houses, and a set of animals, faces, houses, and objects. All animals were 4-legged mammals, presented in different viewpoints. The images from the Stirling/ESRC 3D Face Database (http://pics.stir.ac.uk/ESRC/) and Radboud Faces Database were used for part of the face stimulus set (Langner et al. 2010). The rest of the stimuli came from images downloaded from a web image repository (www.shutterstock.com). The images were preprocessed using a custom MATLAB script. The images were isolated from their backgrounds, converted to grayscale, and resized so that the maximum horizontal or vertical extent for the stimuli was 4°. The mean luminance and contrast across all images of all categories were matched using the SHINE toolbox (Willenbockel et al. 2010). The images were presented on a uniform background (8-bit RGB values: 230, 230, 250; the luminance: 54 cd/m^2^). The perception of the stimuli looking darker at lower SNR levels (Fig. 1B) is due to a higher proportion of salt and pepper noise levels and the relatively brighter background. However, the images of a particular SNR level had the same mean luminance. Thus, luminance could not be used as a cue for the categorization and hence the differences in luminance across SNR levels are highly unlikely to have affected the preferences of the monkeys for a particular category choice. Figure 1B shows examples of the stimuli. We manipulated the signal-to-noise ratio (SNR) of the images by adding “salt-and-pepper” noise: We replaced a fixed percentage of the image pixels and the surrounding 5° by 5° square area with equal numbers of black and white pixels. The SNR corresponds to the percentage of pixels that were not replaced by noise.

### Experimental setup

The position of 1 eye was continuously tracked using an infrared video-based tracking system (SR Research EyeLink; sampling rate: 1 kHz). Stimuli were presented on a CRT display (Philips Brilliance 202 P4; 1,024 × 768 screen resolution; 75 Hz vertical refresh rate) at a distance of 57 cm from the monkey’s eyes. The offset and onset of the stimulus were signaled by a photodiode that detected changes in luminance of a square placed in the corner of the display, which was invisible to the monkey. A digital signal processing-based computer system-controlled stimulus presentation, event timing, electrical stimulation timing, and juice delivery while sampling the photodiode signal, eye positions, stimulus, and behavioral events, which were then stored for offline analysis.

### Behavioral training and tasks

Both monkeys had already been trained on a passive fixation task, where they had to fixate a red dot at the center of the display within a 2–3° fixation window. Later, both monkeys were trained in several 2-choice categorization (2CC) tasks. First, the monkeys were trained to categorize animals versus objects, with animals associated with an upper visual field target (eccentricity of 4°) on the vertical meridian and with objects associated with a lower visual field target of the same eccentricity. The monkeys were then trained to categorize faces versus objects, where the faces were associated with the same target location as the animals. Subsequently, MG was trained to categorize houses versus objects, with houses associated with the same target location as animals and faces. In the subsequent 4-choice categorization (4CC) task, the monkeys were required to categorize exemplars of 4 categories simultaneously. Then, both monkeys were trained for additional 2CC tasks in which they had to discriminate animals versus objects, houses versus objects, faces versus houses, faces versus objects, faces versus animals, and houses versus animals with the spatial locations of the targets for the category choices preserved from the 4CC task. Finally, the monkeys were trained in a selected set of 2CC tasks in which the spatial location of the targets was reversed.

In the 2CC tasks (Fig. 1A), the monkeys fixated on a central red spot for 500 ms after which either a centered animal, face, house, or object image (“cue”; 4° visual angle) was presented for 200 ms, centered on the fixation point. The images with different SNRs were randomly interleaved (for SNR levels, see figures). After an interstimulus interval (ISI) of 13.33 ms, the monkeys were presented with 2 target images, which were drawn from the same image set as the cues and all had an SNR of 100%. The monkeys were required to make a saccade to the target of the same category as the cue. The targets were shown until the monkeys made a saccade, with a maximum time limit of 5,000 ms. The size of the target zone where the gaze was detected as a choice varied between monkeys, ranging between approximately 6° and 9° for the horizontal axis and between approximately 8° and 12° for the vertical axis. The monkeys worked for a fluid reward (apple juice) until they were satiated. The 4CC task (Fig. 1A) was similar except that the monkeys were provided simultaneously with 4 target images. The target images were positioned on the vertical (faces and objects) and horizontal axis (animals and houses). The spatial locations of animal and house targets were switched between monkeys along the horizontal axis to control for an effect of the target location (ipsi—vs. contralateral to the hemisphere receiving EM).

### Recording and EM procedures

The electrophysiological setup was the same as previously used (Kumar et al. 2017). The multiunit and LFP recordings were performed with epoxylite insulated tungsten microelectrodes (FHC). The impedance of the electrodes was lowered to between 0.3 and 1 MΩ. The electrode was slowly advanced into the brain with a Narishige Microdrive through a stainless steel guide tube fixed in a Crist grid that was placed within the recording chamber. Spiking activity was obtained by filtering the amplified signal between 540 and 6 kHz. Multiunit activity (MUA) was recorded online by thresholding the spikes using a custom amplitude discriminator. The simultaneously measured LFPs were filtered online with a 1–300 Hz bandpass filter and were saved for offline analysis.

The Crist grid positions were selected so that the electrode would target the center of the fMRI-defined ASB in both monkeys. We used the fMRI mapping and analyses described previously (Popivanov et al. 2012; Taubert et al. 2015). In MB, we used the previous study’s fMRI data (Popivanov et al. 2012) and the body patch was defined by the contrast monkey bodies minus (matched) objects, excluding voxels that showed an activation to faces versus objects (see Popivanov et al. 2014). The fMRI data of MG are from Taubert and colleagues (Taubert et al. 2015). This monkey was scanned with stimuli of different classes that were identical to those used in a previous study (Tsao et al. 2003). The contrast was bodies (without heads) minus faces, fruits, tools, and hands. The fMRI maps were coregistered with the anatomical magnetic resonance imaging (MRI) of each monkey and the coregistration was verified by visual inspection. For each monkey, body patches were plotted using xjView (v8.14) with a threshold of *t* = 7.0 (note that *t* = 4.9 for *P* < 0.05 familywise error rate). Recording locations (Fig. 1C) along the anterior–posterior and medial–lateral dimensions were extrapolated from the trajectories of MRI-imaged capillaries that contained an electrode inserted in the grid, as given in Kumar et al. (2017). The ventral–dorsal location of the electrode tip was verified in each recording session using the silence that marks the sulcus between the banks of the STS. We recorded and stimulated the right hemisphere in both monkeys. Although we employed different stimulus sets and contrasts to map the body patches with fMRI in the 2 monkeys, their location was similar in the 2 animals and corresponded to ASB as observed in other monkeys and different stimulus sets in other studies (Popivanov et al. 2012; Premereur et al. 2016; Bao et al. 2020; their “middle body patch”). Importantly, we measured the responses (MUA and gamma LFP power) to the same search stimulus set for each penetration in each monkey, thus verifying the location of the electrode in the body-selective patch before EM (see Results).

We employed tungsten electrodes for EM, as in Afraz et al. (2006). According to the manufacturer (FHC), the electrodes we used in this study have a tip size of between 1 and 3 μm. In order to reduce across-session variability in impedance, we used as much as possible the same electrodes across sessions (typically, 5–10 sessions per electrode). A saline solution (0.9% w/v NaCl [308 mOsm/L]) was used to test the impedance before starting each session. We verified the impedance of the electrode again after the penetration before starting the EM and after session completion. In cases where the impedance was outside the range of 0.3 and 1 MΩ before starting the sessions, we replaced the electrode.

During EM, a pulse train of 150 ms was applied during cue presentation, 50 ms later than the cue onset (to account for the response latencies of ASB neurons). A stimulus isolator (WPI, A365) was used to generate the stimulation pulses, driven by a pulse generator (WPI, A310 accupulser), which was triggered for each stimulation train by the TTL output of a custom computer system. A pulse frequency of 200 Hz and current amplitude of 50 or 150 μA was used (see Results). Bipolar, cathodal-first current pulses were charge-balanced, with a phase duration of 250 μs and a distance between the 2 phases of 50 μs.

In each daily session, we recorded MUA and LFPs before performing the EM during the categorization task to measure the category selectivity of the site by presenting the images of the search stimulus set in a pseudorandom order. Images were presented for 200 ms each with an ISI of approximately 400 ms during passive fixation (fixation window size: 2° × 2°), as in a previous study (Popivanov et al. 2014). The images (*n* = 60) were presented randomly interleaved in blocks of 60 unaborted presentations. The ISIs within and between successive blocks were the same. Fixation was required between 100 ms prestimulus and 200 ms poststimulus presentation. Juice rewards were given with decreasing intervals as long as the monkey maintained fixation. Aborted presentations were not analyzed further. Recordings continued until we reached a minimum number of 5 unaborted presentations for each image.

#### EM in 2CC tasks

We performed the EM experiments after the recordings in each daily session. Neurons were stimulated at the same depth with the same electrode as in the preceding recordings, i.e. without moving the electrode. Three 2CC tasks were tested: (i) animals versus objects, (ii) faces versus objects, and (iii) houses versus objects. We used 300 animal and 300 object images for the “animals versus objects” categorization task. For the other 2 tasks, 225 images were available for each category. The object images differed among the 3 tasks to minimize differences in low-level stimulus features (e.g. aspect ratio) between the objects and the paired categories. For each session, a different image set was created with 70 images per category in the case of the “animals versus objects” task and with 50 images per category in the case of the other 2 tasks. Of the 70 or 50 images per category, 30% were images presented during the last 5 daily sessions, while the remaining 70% were images that had not been shown to the monkey during the last 5 preceding daily sessions. The cue images were presented at 4 SNRs in a randomly interleaved order. The SNRs employed in a particular session were titrated to the monkey’s performance and depended on the categorization task. The noise pattern varied from 1 trial to another. EM of 150 μA was applied in 50% of the trials in random order.

#### EM in 4CC tasks

As with the 2CC task, EM was performed immediately after the MUA/LFP recordings without moving the electrode. A total of 300 images per category were used. In each daily session, an image set was created using 50 images per category. Of these 50 images per category, 30% were presented during the last 5 daily sessions, while the other 70% of the images had not been shown to the monkey during the last 5 sessions. EM of 50 μA was applied in 50% of the trials. The cue images were presented at 4 SNR levels in random order. The SNR levels used in a particular session were adjusted according to the monkey’s performance.

#### EM in 2CC tasks with different target locations

These 2CC tasks were presented after completion of the 4CC task. They differed from the earlier 2CC tasks by the spatial location of the target stimuli. The 2 targets could be presented not only on the vertical meridian but also could be presented perpendicular to each other or on the horizontal meridian. The location of the 2 saccade targets for each 2CC task was initially identical to that in the 4CC task. Six different categorizations were tested in individual sessions: (i) animals versus objects, (ii) houses versus objects, (iii) animals versus faces, (iv) faces versus houses, (v) faces versus objects, and (vi) animals versus houses. Afterward, and following retraining, the spatial location of the targets was reversed to those in the 4CC task (left–right reversal). For the 2CC tasks, 300 images were available for each category. For each daily session, we created a new image set of 50 images per category. As before, 30% of the images had been shown during the last 5 sessions, while the remaining 70% had not been shown during the last 5 sessions. The cue images were presented at 4 SNR levels. The SNR levels employed in a session were adapted to the monkey’s performance for the category pair. EM of 50 μA was applied in 50% of the trials.

The order of the different tasks for each monkey is shown in Supplementary Table S1. We adhered to the following rule: For each negative finding of a 2CC task, i.e. absence of a behavioral effect due to EM, we repeated and replicated the previous positive findings of the other 2CC task. This ensured that an absence of a behavioral effect of EM does not result from the repetition of EM, as the latter may decrease the behavioral effect of EM (Salzman and Newsome 1994). Sample sizes were based on previous experiments using similar methods.

### Data analysis

#### MUA

The firing rate of each unaborted stimulus presentation was determined in 2 analysis windows: a baseline window ranging from 100 to 0 ms before stimulus onset, and a response window ranging from 50 to 250 ms after stimulus onset. All analyses were based on baseline subtracted, averaged net firing rates. For each site, the response to a stimulus was normalized by dividing the mean net firing rate for that stimulus (averaged across presentations for that stimulus) by the response to the stimulus with the highest mean firing rate. Then, for each site, we averaged the normalized responses across the different monkey body and nonprimate mammal images to obtain a body category response. Likewise, we averaged the responses to the 20 object images and 10 face images to obtain responses for the nonbody category. For each recording/stimulation site, we computed a body selectivity index (BSI; Popivanov et al. 2014), defined as follows:

$$ \mathrm{BSI}=\frac{{\overline{R}}_{\mathrm{body}}-{\overline{R}}_{\mathrm{non}-\mathrm{body}}}{\mid{\overline{R}}_{\mathrm{body}}\mid +\mid{\overline{R}}_{\mathrm{non}-\mathrm{body}}\mid }, $$

where ${\overline{R}}_{\mathrm{body}}$ and ${\overline{R}}_{\mathrm{non}-\mathrm{body}}$ were the mean net firing rates (MUA) to bodies and nonbodies of the category set stimuli, respectively.

#### High gamma LFP power

We employed the same procedures to compute the high gamma LFP power as previously described (Popivanov et al. 2014). Briefly, single-trial LFP data were convolved using complex Morlet wavelets and the square of the convolution between wavelet and signal was taken. The Morlet wavelets had a constant center frequency and a spectral bandwidth ratio (f0/σf) of 7. The mean power across presentations of a stimulus per spectral frequency and per site was computed. The power was normalized by dividing it by the average power in a baseline window ranging from 100 to 0 ms before stimulus onset. For each stimulus, the high gamma LFP power was computed by averaging the mean normalized power in a 50–250-ms window, relative to stimulus onset, between 60 and 150 Hz. LFP power in this high gamma band can be used as a proxy for the spiking activity of the neural population surrounding the electrode (Ray and Maunsell 2011). For quantitative analyses of the mean power across sites, the contribution of each site to the population response was equated by dividing the power by the maximum power across the 60 images for each site. These power values were then averaged across the images of the body (monkey bodies and mammals), face, and object categories.

#### 2CC performance

The performances with and without EM in the “animals versus objects” categorization task were computed as the proportion of animal choices as a function of the SNR for animal and object cue images separately. To create a continuum of stimulus noise levels, SNRs were given a positive sign for animals and a negative sign for objects. We will label these signed SNRs as “stimulus levels.” Thus, an animal image with an SNR of 40% has a stimulus level of 40%, while an object image with an SNR of 40% has a stimulus level of −40%. The same procedure was applied to other categorization pairs, e.g. for “faces versus objects,” the face images were assigned positive values and the percentage of face choices was computed.

To quantify the shift of the psychometric curves due to EM, logistic functions were fitted to the monkey’s choices with the following function:

(1) \begin{equation*} P\left(x;\alpha, \beta, \lambda \right)={\left[1+{e}^{-Q}\right]}^{-1},Q=\alpha +\beta x+\lambda I, \end{equation*}

where *x* is the stimulus level of the cue, and *P(x)* is the proportion of animals, face, or house choices (depending on the categorization task). The dummy variable *I* indicates the presence or absence of EM, and α and ${\beta}$ are free parameters measuring the choice bias and slope of the psychometric curve, respectively. The 𝜆 parameter measures the effect of EM on the monkey’s choice bias. The normalized shift of the psychometric function was defined as the change in stimulus level that would have induced a behavioral effect equal to that of the EM. This is equal to 𝜆/${\beta}$ in the logistic fit (DeAngelis et al. 1998; Afraz et al. 2006; Verhoef et al. 2012). For fitting the models, an iterative nonlinear least-squares algorithm (MATLAB 2015b, “lsqnonlin” function with “multistart” algorithm) with an objective to minimize the sum of squared differences between observed and predicted choices was implemented. The fit was performed separately for the behavioral data obtained in each session and for all the sessions pooled together. A null distribution of 𝜆/${\beta}$ was created by randomizing 1,000 times the EM and non-EM trials per SNR level, then splitting the randomized data into 2, which was followed by fitting the psychometric curves. This randomization test examined whether the shift between the EM and non-EM curves was significantly different from 0. The randomization test was performed for the data from individual sessions or the pooled data of all sessions of a task. The effect of EM was considered to be significant when the percentile of the obtained 𝜆/${\beta}$ was >97.5 of the null distribution. In the figures, we present the 𝜆/${\beta}$ (shift) values computed for the data pooled across multiple sessions of the same task. The confidence intervals (CIs) were computed using a bootstrapping approach. We sampled, with replacement, trials of the EM and non-EM conditions. Using the same procedure as outlined above, we computed the shift value based on the resampled data. This was repeated for 1,000 times and the resulting distribution was employed to compute the 95% CI using the percentile method.

For illustrative purposes, we show in the figures psychometric functions fitted to the data using the following equation:

(2) \begin{equation*} P\left(x;\alpha, \beta, G,L\right)=G+\left(1-G-L\right){\left[1+{e}^{-Q}\right]}^{-1},Q=\beta \left(x-\alpha \right), \end{equation*}

where parameters α and β correspond to the threshold and slope of the psychometric function, respectively, and *G* and *L* correspond to guess and lapse rates, respectively.

The change in slope (sensitivity) of the psychometric curves was assessed using the following function:

(3) \begin{equation*} P\left(x;\alpha, \beta, {\lambda}_1,{\lambda}_2\right)={\left[1+{e}^{-Q}\right]}^{-1},Q=\alpha +\left(\beta +{\lambda}_1I\right)x+{\lambda}_2I, \end{equation*}

where *x* is the stimulus level of the cue, and *P*(*x*) is the probability of the category choice (Fetsch et al. 2014). Definitions of the parameters are the same as above, where *I* indicates the presence or absence of microstimulation, and α, ${\beta}$, 𝜆_1_, and 𝜆_2_ are free parameters. The α parameter measures the choice bias, whereas ${\beta}$ and 𝜆_1_ jointly represent the slope of the psychometric curve. The 𝜆_2_ parameter measures the effect of EM on the monkey’s choice bias. The change in the slope of the psychometric curve is represented by parameter 𝜆_1_. A randomization test was performed (1,000 randomizations) to determine whether the change in slope was significantly different from 0.

#### 4CC performance

For each monkey, we assessed whether EM affected the overall categorization performance (sensitivity) by plotting the proportion of correct choices as a function of SNR of the cue with and without EM. The chance level was 25%. Also, the proportion of choices of each of the 4 target categories (animals, faces, houses, and objects) was plotted as a function of the SNR of each of the cue categories, producing 16 curves (4 cue categories × 4 choices) with and without EM. Then, we averaged the proportion of choices of a particular category across the 4 cues, producing 4 curves, i.e. 1 for each choice category, with and without EM. A polynomial order 2 (quadratic) function was fitted to the proportion of the monkey’s choices for each category with the “polyfit” (MATLAB 2015b) function, separately for each of the 4 choice categories and EM conditions.

For each monkey, we equated the number of trials for the data obtained with and without EM and for the different stimulation sites for comparative analysis. We used a randomization test to determine if the effect of EM was significantly different from 0. For each of 10,000 randomization runs, the combined EM and non-EM data were randomly split into 2 halves and a second-order polynomial function was fitted to the means of the proportion of choices of each category as a function of SNR (8 means). Then, the mean squared error (MSE) was computed across the 4 SNRs. MSEs of the randomization runs were used to generate a null distribution and the difference between EM and non-EM trials was considered to be significant when the percentile of the observed MSE was >0.9875 (*P* < 0.0125; Bonferroni corrected for 4 choice categories).

### Controls

#### Generalization test

Generalization across category exemplars, which is an essential feature of categorization, was tested in the 4CC task for both monkeys. This test included 50 images from each category of which 40% were novel, i.e. never presented before. Based on the previous performance in the 4CC task, images were presented with an SNR of 80% and 60% for MB and MG, respectively. No EM was delivered during the generalization test. Only the first presentation choices of the stimuli were used for data analysis. The performance of both monkeys did not differ significantly between the old and novel images on the very first presentation of an image in the session (Fig. 1E; Chi-Square test with Yates’ Continuity correction; *P* = 0.81 and *P* = 0.21). This shows that the performance of the monkeys was not merely determined by rote learning of exemplars and that they were able to categorize novel images of the 4 categories.

#### Contribution of low-level image features

One advantage of low SNR images of which the noise pattern varies across trials is that it discourages the use of local, low-level stimulus features. To further examine the potential contribution of low-level features to the categorization, we presented a randomly selected subset of 109 images per category (animals, faces, objects, and houses) from the 4CC task to a deep convolutional neural network, AlexNet (Krizhevsky et al. 2017). We employed the default Alexnet implementation in Matlab that had been trained to classify approximately 1.2 million natural images divided into 1,000 classes for the ImageNet Large Scale Visual Recognition Challenge 2012 (Russakovsky et al. 2015). We trained a linear Support Vector Machine with 5-fold crossvalidation on the activation of the units of the first convolutional layer, conv1, to classify the images of the 4 categories at each SNR. The classification score is the mean performance across the 5-fold (chance level = 25% correct). The classifier, using the Alexnet’s first layer activations, performed markedly worse than our monkeys (Fig. 1D), suggesting that low-level features cannot account for the monkeys’ performance in the categorization task. This is also supported by the excellent first-trial generalization performance for completely novel images that differed in local, low-level features from previously seen images (Fig. 1E).

## Results

We used fMRI mapping to localize the body-selective patch ASB in the lower bank of the anterior STS (Fig. 1C). For recordings and EM, we targeted ASB locations with strong fMRI-defined body-selective activations (*t* > 7.0). Body category selectivity was confirmed with MUA and local field potentials (LFP) recordings in the ASB for each monkey using our standard stimulus set (Popivanov et al. 2014). As expected, the body patch sites responded stronger to images containing bodies (monkey bodies and 4-legged mammals) than to objects and human faces. This was confirmed for both MUA (bodies vs. objects and bodies versus faces (1-sided Wilcoxon signed-rank test; all *P* < 0.001) and high gamma (60–150 Hz) LFP power (all *P* < 0.001) at 2 sites in each monkey. The responses to objects and faces were statistically indistinguishable (all *P* > 0.05), although there was a tendency in monkey MB for higher response to faces compared to objects. Note that these are electrophysiological data from the same sites later stimulated (EM) in the same session. Below, we will use high gamma LFP as metric of neural selectivity of the stimulated site as it reflects the activity of a wider region (radius estimated to be at least 250 μm; (Jia et al. 2011; Ray and Maunsell 2011) than MUA, but MUA showed similar selectivity trends.

### EM in 2CC tasks

**Fig. 2:**
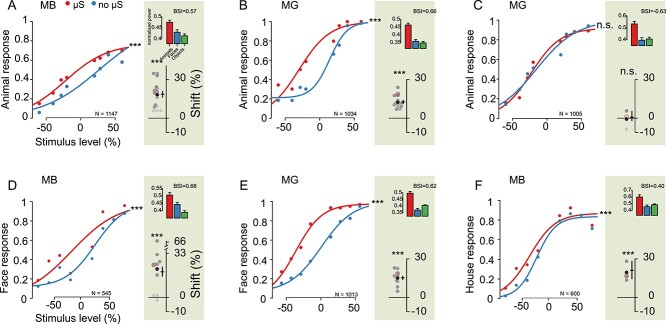
Effect of microstimulation on the performance in 2CC categorization tasks. The proportion of animal, face, or house choices in the animals versus objects A–C), faces versus objects D and E), and houses versus objects F) 2CC tasks, respectively, are plotted as a function of stimulus level. The red and blue dots represent the stimulated (μS) and nonstimulated (no μS) conditions, respectively. *N* = total number of trials. Significance of the difference between the stimulated and nonstimulated conditions are indicated by symbols: ^*^^*^^*^  *P* < 0.001; n.s. *P* > 0.05. A, B) EM effect for the animals versus objects task in locations inside ASB. The negative stimulus levels represent the SNR levels of the nonanimal images, while the positive values represent the SNR levels of the animal images. A) Data of MB and B) data of MG. Left: Single session examples corresponding to the red circle in the inset distribution. The curves are a logistic fit (Equation (2) of Materials and methods). The top plots of the insets (shaded region) show the mean normalized high gamma LFP power for animals, faces, and objects measured at the sites in the same sessions preceding the EM task. Error bars represent standard errors. In conjunction with the LFP plots, the BSI calculated from the MUA is also shown. The bottom plots of the insets show the fitted shift values (𝜆/${\beta}$, fitted Equation (1) of Materials and methods), which were computed for all individual sessions. Positive shift values correspond to an increased proportion of animal choices with EM. Significant shift values are indicated by a dark gray color (*P* < 0.05). The mean shift is indicated by the black-filled circle. Horizontal back bars indicate the shift computed for psychometric functions that were fitted to the data pooled across all sessions. Vertical error bars represent 95% CIs, which were computed by bootstrap resampling. The symbols above the distributions indicate the statistical significance of the shift of the pooled psychometric functions, which was assessed with a randomization test (see Materials and methods). C) Performance of MG in the animals versus objects task with EM for a neighboring location in ASB. Same conventions as in A). D and E) EM effect for the faces versus objects task for locations inside ASB. Positive shift values correspond to an increased proportion of face choices with EM. Same conventions as in A). D) Data of MB and E) data of MG. F) EM effect for the houses versus objects task for a location inside ASB (MB). Same conventions as in A). Positive shift values correspond to an increased proportion of house choices with EM.

Both monkeys were trained to perform a 2CC task (Fig. 1A) of animals versus objects. They were required to make an upward saccade to a target after the presentation of an animal image and a downward saccade for an object image. A wide variety of images for each category were presented as cues at different SNRs. Initially, we assessed the effect of 150 μA EM of sites that responded stronger to mammals than objects and faces. EM was applied in 50% of the trials for 150 ms during the 200 ms cue presentation with a 50-ms delay relative to cue onset. EM effects differed for 2 adjacent grid positions in monkey MG, although both sites were inside the fMRI-defined ASB and demonstrated body category selectivity in high gamma power (Fig. 2, insets) and MUA. We will present the data of the 2 ASB sites of this monkey separately, although pooling the data of the 2 sites did not alter the main result (see below).

EM of an ASB site increased the proportion of animal choices. This is shown for each monkey for an example session in Fig. 2A and B. We quantified the change in choice bias due to EM by computing the shift of logistic functions fitted to the psychometric data of the EM and non-EM conditions (see Materials and methods). This was done for each session and pooled data across sessions (Fig. 2A and B). Figure 2 shows that the shifts over sessions (black-filled circles) were similar to the shifts for the mean psychometric curves of the data pooled (horizontal black bars in Fig. 2). Each of the EM sessions (MB: 16 sessions; MG: 15 sessions) resulted in a shift toward animal choices. A randomization test showed that the proportion of animal choices increased significantly (*P* < 0.001 for each monkey on pooled data across sessions; Fig. 2). EM did not change the slope of the psychometric curves (see Materials and methods, *P* > 0.28 for each monkey). This can also be appreciated by comparing the across-session averaged percent correct, plotted as a function of SNR (Supplementary Fig. S1), for the EM and non-EM trials. Thus, EM increased animal choices but did not affect the categorization accuracy per se. The absence of an effect on the categorization accuracy suggests that the shift of the psychometric function is not caused by a general interference effect of EM. Moreover, EM did not affect the proportion of premature saccades, i.e. saccades made before target onset (Supplementary Table S1). We observed small differences in RTs between the EM and non-EM conditions in the 2CC task (Supplementary Fig. S3), but these were inconsistent across the 2 monkeys despite the consistent effects for the choice frequency. However, our task was not designed for capturing true reaction times because the monkeys had to wait until the target onset before initiating a saccade. Hence, a simple interpretation of the reaction times is not possible.

We found no EM effect at a nearby ASB location (grid position 1 mm away) in MG (Fig. 2C; randomization test; *P* = 0.38; data pooled across 4 sessions). The absence of an EM effect at this grid position did not result from a fewer number of trials, as we observed a significant shift in the proportion of animal choices at the other position when equating the number of trials for the 2 positions. Since we tested this grid position before the one that had an EM effect (Supplementary Table S2), the lack of an EM effect did not reflect a reduction of the EM effect over time. This variation of the EM effect for these nearby grid positions may suggest heterogeneity in the body patch readout at millimeter scales. However, the categorization accuracy also differed for the 2 positions, and thus it cannot be excluded that differences in categorization strategy underlie the discrepant EM effects. Note that also when pooling the data of the 2 grid positions, the EM effect in MG is still highly significant (shift = 0.11, *P* < 0.001; Bootstrap CIs = [0.096 0.126]).

Next, we examined the category specificity of the EM effect at the ASB locations, which showed an increased proportion of animal choices in the animal versus object categorization. We trained the monkeys in a faces versus objects categorization task in which they were required to saccade upward for faces and downward for objects. EM increased in the proportion of face choices (Fig. 2D and E; *P* < 0.001 in each monkey), with a shift of the psychometric function similar to that for animal categorization. The high gamma LFP power, measured at the same locations before EM, was stronger for faces compared to objects in MB but the opposite trend was present in MG (Fig. 2D and E, insets). Hence, the EM effect for face categorization differed from the neural selectivity measured at that site.

ASB is close to a face patch (AL) and it cannot be excluded that the EM effect arises from current spreading to the face patch. Therefore, we trained MB to categorize houses versus objects. Houses form a relatively homogeneous category, like faces, and human fMRI studies showed that houses activate the parahippocampal place area (Epstein and Kanwisher 1998), which is distant from body category-selective regions. Furthermore, houses, relative to objects, do not activate the rostral STS where ASB is located (Moeller et al. 2017). In the houses versus objects categorization task, the house choice was associated with the same target location as the animals and faces in the preceding categorization tasks. Surprisingly, EM during the house categorization increased the proportion of house choices (Fig. 2F; *P* < 0.001).

### EM in 4CC task

In the above experiments, animals, faces, and houses were linked to an upward saccade. It is plausible that the increased proportion of face and house choices with EM was an increase in animal choices since the monkeys were initially trained to make that choice for the animal category. Hence, EM might have induced a body signal that produced a behavioral choice that was associated with animals, i.e. an upward saccade. Another possibility is that the monkeys performed an object versus nonobject categorization instead of an object versus animal/face/house categorization. When EM produced a body-like percept, this could have increased the nonobject choices even in the case of faces versus objects or houses versus objects categorizations (faces and houses are nonobjects, like bodies). We think this possibility is unlikely since the object category is more heterogeneous than the body, face, and house categories, but it cannot be excluded. A third possibility for the EM effects in the 3 different tasks is that EM of ASB does not show category selectivity. To examine these possibilities, we trained the monkeys in a 4CC task in which saccade directions were unique to each category (animals, faces, houses, and objects; Fig. 1A), which were randomly presented within a session.

Alternatively, the EM effect lacks category selectivity. To dissociate these possibilities, we trained the monkeys in a 4CC task in which saccade directions were unique to each category (animals, faces, houses, and objects; Fig. 1A), which were randomly presented within a session. EM was delivered in 50% of the trials, but we decreased the current to 50 μA to reduce the current spread. The cue images were presented with SNR levels of 20%, 40%, 60%, and 80% and 10%, 25%, 40%, and 60% to MB and MG, respectively.

**Fig. 3:**
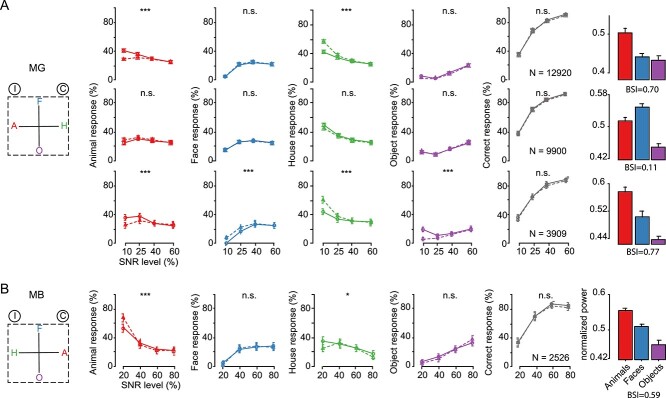
Effect of microstimulation on the performance in the 4CC task. Left (first) column: The insets show the spatial location of the choice targets for 4 categories: animals (A), faces (F), houses (H), and objects (O). I and C indicate the hemifield ipsilateral and contralateral, respectively, to the stimulated hemisphere. The spatial locations of animal and house targets were switched between the monkeys along the horizontal axis. Second to fifth columns: The effect of EM on the animal (second column), face (third column), house (fourth column), and object (fifth column) choices, plotted as a function of SNR. Sixth column: Percent correct choices plotted as a function of SNR. *N* = number of trials. The full lines (and circles) and dotted lines (and triangles) represent the nonstimulated and stimulated conditions, respectively. Error bars represent 95% CIs for proportions. Right (seventh) column: Mean normalized high gamma LFP power for animals, faces, and objects before the corresponding EM sessions. Error bars represent standard errors. A) Top row: Effect of low current EM (50 μA) inside ASB for MG. Second row: Effect of low current EM (50 μA) outside ASB for MG. Third row: Effect of high current EM (150 μA) inside ASB for MG. B) Effect of low current EM (50 μA) inside ASB for MB. Same conventions of the symbols for statistical significance as given in Fig. 2.

Consistent with the 2CC task, the categorization accuracy was not affected by EM (Fig. 3). However, EM induced shifts in the choices, which can be appreciated when averaging the choices for a particular category across the 4 cue categories for each SNR (Fig. 3). EM in ASB of MG significantly decreased the percentage of animal choices (randomization test; *P* < 0.001; Fig. 3A; upper row). Concomitantly, the percentage of house choices increased (*P* < 0.001). These effects were present mainly for the low SNR levels. No effect for face (*P* = 0.55) and object choices (*P* = 0.14) was present in this monkey. The decrease in animal choices in the 4CC task in MG contrasts with an increase in animal choices in the 2CC task (Fig. 2B). In the 2CC experiments, 150 μA was employed, thus we also performed EM sessions with the 4CC task in MG using 150 μA. We again observed a decrease in animal choices and an increase in house choices (both *P* < 0.001), but there was also a decrease in object choices (*P* < 0.0001) and an increase in face choices (*P* = 0.0001; Fig. 3A, lower panel). Thus, the effect of 150 μA EM was present for all choice categories, with the house and animal choices affected as for 50 μA.

In contrast to monkey MG, EM using 50 μA in MB increased animal choices significantly (*P* < 0.001, Fig. 3B; upper row) at low SNR levels. The house choices significantly decreased in frequency (*P* = 0.017). As in MG, no EM effects for the face (*P* = 0.55) and object (*P* = 0.24) choices were present, although this may have resulted from the strong choice bias of MB against those choices for the low SNR condition.

Thus, EM (50 μA) affected animal choices in the 4CC task, although in opposite ways in the 2 monkeys. No reliable effect of EM was present on the overall performance level (see plots of percent correct as a function of SNR; right column of Fig. 3). To examine whether the EM effect on the animal choices was region-specific, we applied EM (50 μA) in MG at an STS site 5 mm away from the ASB stimulation locations. At that site, the high gamma LFP power was similar for bodies and faces but was greater than for objects and the BSI of the MUA was only 0.11 (Fig. 3A; rightmost panel). Stimulating this site had no significant behavioral effect (Fig. 3A; middle panel). Note that we used the same number of trials for the different sites for the statistical testing. Also, the sites inside and outside ASB were tested interleaved (Supplementary Table S1) to control for order effects. These data show that the effect of EM on the choices in the 4CC task depends on the stimulated STS region.

**Fig. 4:**
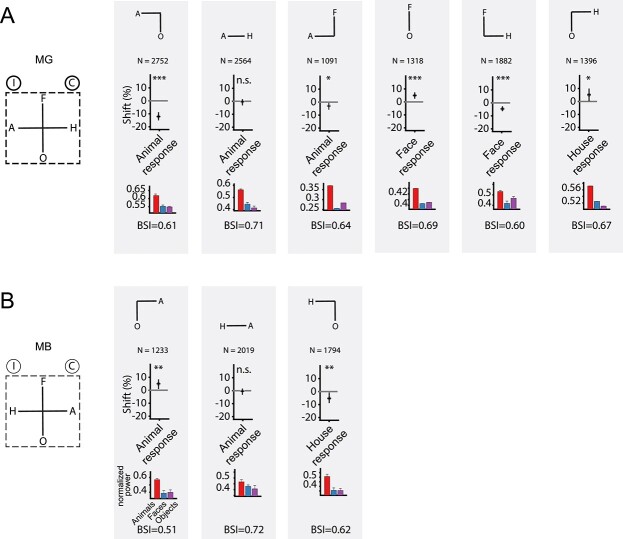
Effect of microstimulation for the 2CC tasks with the same target locations as for the 4CC task. First column: Spatial locations of the choice targets. Same conventions as in Fig. 3. Second to seventh columns: Top: Spatial locations of the targets used in the corresponding 2CC tasks for MG A) and MB B), which were preserved from the 4CC task. *N* = number of trials. Middle: Fitted shift values (and 95% CIs) for the data pooled across all sessions in the animals versus objects (second column), animals versus houses (third column), animals versus faces (fourth column), faces versus objects (fifth column), faces versus houses (sixth column), and houses versus objects (seventh column) categorization task in MG. Positive shift values correspond to an increased proportion of choices, with EM, of the image category shown below the abscissa (e.g. for the animals vs. objects task [second column], an increase of animal choices). Symbols indicate statistical significance: ^*^  *P* < 0.025; ^*^^*^  *P* < 0.01; ^*^^*^^*^  *P* < 0.001; n.s. *P* > 0.05. Bottom: Mean normalized high gamma LFP power for animals, faces, and objects measured at the sites in the same sessions preceding the EM. Same conventions as in Fig. 2. B) Effect of EM (50 μA) for the animals versus objects (second column), animals versus houses (third column), and houses versus objects (fourth column) tasks in MB. Same conventions as in A).

### EM in 2CC tasks with target locations as in 4CC

In the 4CC task, EM of ASB consistently affected choices for animals and houses categories. To assess how these were related (e.g. an increase in animal choices inducing a decrease in the house choices, or vice versa), pairwise testing of different categories was conducted: the 6 possible pairs in MG and 3 pairs (animals vs. objects, houses vs. objects, and animals vs. houses) in MB. The target locations for these 2CC tasks were the same as for the 4CC task, with 2 instead of 4 targets. For example, in the “houses versus objects” 2CC task for MG, the house targets were presented to the left, while the object targets were at the display’s bottom (Fig. 1A). Supplementary Table S2 presents the order of the different 2CC tasks. Using the same EM sites from the 4CC task, a 50 μA current was applied. The SNRs were selected based on the training performance.

Consistent with the results of the 4CC task, EM in the animals versus objects 2CC task decreased animal choices in MG (Fig. 4A; *P* < 0.001; pooled across sessions) and increased animal choices in MB (Fig. 4B; *P* = 0.008). Additionally, there was a trend toward a reduced proportion of animal choices for animals versus faces (only tested in MG; *P* = 0.035; Fig. 4A). However, the inclusion of bodies in the categorization task did not guarantee a behavioral effect of ASB EM: in both animals, EM failed to produce an effect when pairing bodies with houses (MG: *P* = 0.38; MB: *P* = 0.37; Fig. 4).

Contrary to the hypothesis that ASB contributes only to categorizations involving bodies, EM in MG resulted in significant shifts in choices in the faces versus houses (Fig. 4A; *P* < 0.001), faces versus objects (*P* < 0.001), and in a trend in the houses versus objects task (*P* = 0.03). Similarly, EM tended to affect choices in the houses versus objects 2CC task in MB (Fig. 4B; *P* = 0.01). Furthermore, in MG, the EM effect for faces was dependent on the other nonbody category: increased face choices when faces paired with objects but decreased face choices when paired with houses (Fig. 4A).

In conclusion, ASB EM can have behavioral effects with or without bodies, but, depending on the paired category, EM effects could be absent even for bodies.

### EM in 2CC tasks with spatially reversed target locations

**Fig. 5:**
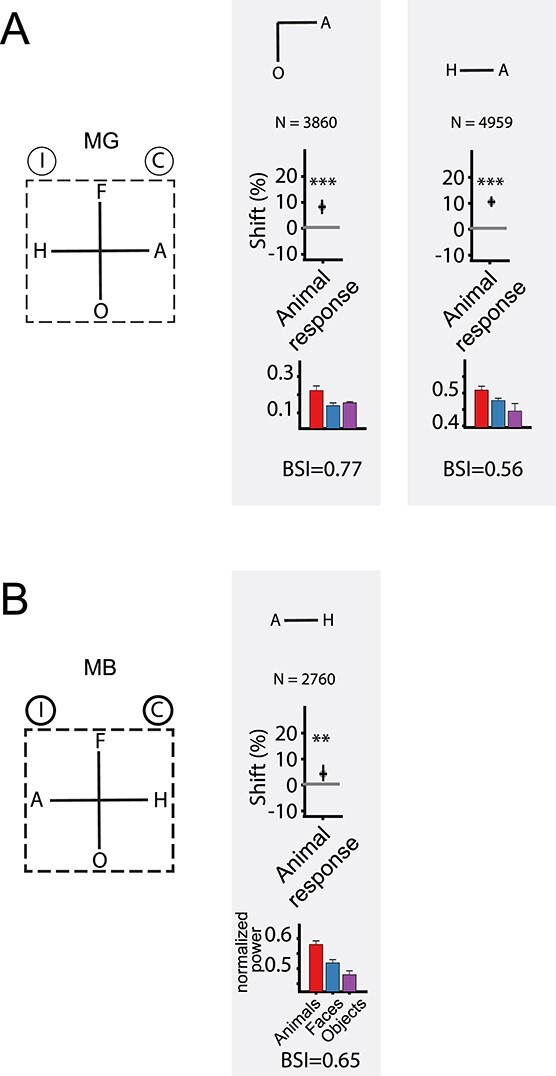
Effect of microstimulation for the 2CC tasks with spatially flipped choice targets. A) MG and B) MB. Effect of EM in the 2CC task, with the spatial location of the animal and house targets being flipped along the horizontal axis (first column). Same conventions as in Fig. 4.

Similar to the 4CC task, the EM effects in the subsequent 2CC tasks were of the opposite sign in both monkeys when animals or houses were included (e.g. animals vs. objects: increase of the proportion of animal choices with EM in MB but the opposite in MG), except for the null result for the animals versus houses categorization. The animal and house targets were in opposite hemifields for both monkeys (Fig. 4). The discrepant effects for the 2 monkeys can be explained when EM increased contralateral choices. To test this, we reversed the location of animal and house targets to dissociate the target location from category choice. In this “reversed” task, the animal target was presented on the left instead of on the right side of the display for MG, and it was vice versa for MB (Fig. 5).

After retraining MG to perform the “reversed” 2CC tasks, animal choices increased with EM of ASB (*P* < 0.001; Fig. 5A). Hence, EM increased contralateral choices in the 2CC task before and after reversal, reconciling the data of the 2 monkeys. However, the hypothesis that EM of ASB merely increases contralateral choices conflicts with the absence of an EM effect for the animals versus houses 2CC task before the reversal in both monkeys (Fig. 4). The reversal of the animal and house targets increased animal choices in both monkeys (*P* < 0.001; Fig. 5A and B). The fact that both monkeys showed a similar behavior despite opposite target locations again argues against the hypothesis that ASB-EM effects were merely a result of increased contralateral choices.

**Fig. 6:**
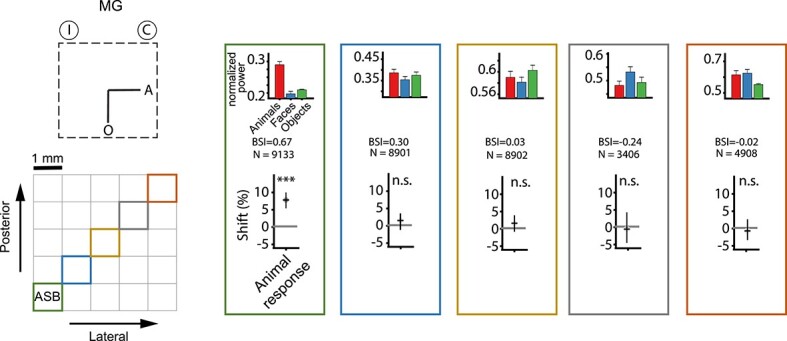
Location dependency of the behavioral effect of EM. First column: Top: Spatial location of the choice targets for the animals versus objects 2CC task. Bottom: 5 different equidistant locations on the recording grid to examine the location specificity of the behavioral effect induced by EM (50 μA; MG). Lateral and posterior dimensions are in grid coordinates. Second to sixth columns: Effect of EM on behavioral performance in the 2CC task. The colored panel borders indicate the corresponding recording grid locations as shown in the bottom panel of the first column. Same conventions as given in Figs 4 and 5.

**Fig. 7:**
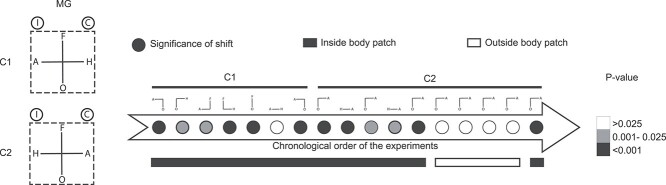
Chronological order and experimental details of the 2CC EM experiments (50 μA; MG). The gray arrow indicates the temporal order of the experiments. The significance level of the behavioral effect of EM is indicated by the gray level of the circles in the arrow (see the legend on the right side). Each circle indicates a different condition and can correspond to multiple successive daily sessions. The filled and open horizontal bars below the arrow indicate that EM was inside or outside of ASB, respectively. First column: Top: Spatial locations of the choice targets (C1). Same conventions as in Fig. 5. Bottom: Spatial location of the animal and house targets being flipped along the horizontal axis (C2).

Above, we showed that behavioral effects depended on the EM location in the 4CC task (Fig. 3). Most IT neurons have a contralateral visual field bias (Op de Beeck and Vogels 2000) and it is unclear why a putative contralateral behavioral bias might be affected by EM location on an millimeter scale. To further test the location specificity of the EM effect, we examined the effect of 50-μA EM on the reversed animals versus objects 2CC task in 5 different locations within the STS lower bank (Fig. 6) of MG. Locations outside the fMRI-defined ASB did not show body-category selectivity of the high gamma LFP power and low BSIs of the MUA (insets in Fig. 6). Only the original ASB location showed a significant EM effect (*P* < 0.001). The absence of an EM effect at the non-ASB locations is not due to order effects since EM stimulation at the original ASB location after testing the non-ASB locations resulted in a significant effect (*P* < 0.001; Fig. 7). Thus, the EM effect in the animals versus objects 2CC task was location-specific on an millimeter scale in IT.

Note that the behavioral changes induced by EM were reliable and consistent across multiple sessions. In the case of the second 2CC tasks, performed after the 4CC task, fewer sessions were run per condition (Supplementary Table S1), preventing across-session assessment of the reliability of the EM effect for some conditions. However, pooling of the data within or across the available sessions showed reliable effects (the number of trials being the number of independent observations). The absence of an EM effect (e.g. for houses vs. animals) was not due to a reduced EM effect with repeated stimulation since subsequent EM sessions replicated the previous effects, as shown in Fig. 7 for MG. Additionally, we interleaved tasks to control for possible order effects of repeated EM in MB (Supplementary Table S2). For some tasks, both monkeys showed choice biases when not stimulated (for instance, for the animal and house targets in the 4CC task). However, the EM-induced shifts in the later 2CC tasks were uncorrelated with the non-EM choice biases (Supplementary Fig. S2). The EM effects did not result from eliciting saccadic responses per se, because, depending on the 2CC task, EM increased or decreased saccade frequency toward a particular target. For instance, EM in MG increased downward saccades for the animals versus objects task, while for the faces versus objects task downward saccades decreased. Additionally, the EM effects were independent of categorization accuracy in the 2CC conditions (Supplementary Fig. S2).

## Discussion

We found that EM in the STS body patch ASB affected the performance in tasks requiring the monkey to categorize complex images under high noise conditions. EM effects were present not only for the category of animals but also for inanimate categorizations such as houses versus objects and faces versus objects. Furthermore, for some but not all categorization tasks, we found an interaction with the visual field location of the saccade targets, with a decreased probability of choices for the targets located ipsilaterally to the stimulated hemisphere. The EM effect was location-specific within the STS on an millimeter scale with the strongest effect on animal categorization occurring in the ASB (tested in 1 monkey). Our findings demonstrate that the behavioral effects of EM in a category-selective patch in a visual categorization task not only reflect the category but can also interact with the task context and choice target location.

In the 4CC and most 2CC tasks, the behavioral sensitivity was not significantly affected by EM, even with 150 μA currents. Previous studies showed impairments in discrimination tasks with relatively high EM currents in IT (face individuation, Moeller et al. 2017); coarse orientation discrimination, Adab and Vogels 2016) and other visual areas (e.g. MT: direction discrimination (Murasugi et al. 1993; Fetsch et al. 2014). In these studies, however, the task required discrimination of feature values (e.g. faces, orientations, or motion directions) that are represented within the region affected by EM. Instead, we employed a categorization task in which monkeys had to discriminate between features represented in the body patch and those represented more in unstimulated parts of IT. This is similar to the low current EM of e.g. 1 direction column in a direction discrimination task, which biases the choice of the monkey but has little to no effect on sensitivity.

We found that EM in the body patch could affect also categorizations that included only nonbody images. This may suggest that activity in the body patch also contributes to the nonbody categorizations. This does not contradict electrophysiology since ASB neurons respond to nonbody images (Kumar et al. 2017) and can categorize, at least to some extent, nonbody categories, for instance, human versus monkey faces (Kumar and Vogels 2019). An alternative explanation is that body patch EM results in body feature signals (e.g. “bodyphenes”) that combine with the representation of weak nonbody signals under noisy conditions, resulting in a behavioral effect at low SNRs. The combination of body signals and nonbody representations is plausible since EM in a human face-category selective area can result in face percepts on top of a simultaneously presented object (Schalk et al. 2017; Jonas et al. 2018). EM in human ventral temporal cortex has also been reported to produce a body “hallucination” (Puce et al. 1999). Another explanation for some of the category-aspecific effects of EM in ASB could be the possibility that the EM is affecting passing axons of other IT subregions such as nearby face patches (HIsted et al. 2009). This is a general drawback of EM experiments. However, another recent study reported that even at a higher current of 300 μA, only nearby voxels (within a few mm) of the electrode are modulated primarily by the EM (Moeller et al. 2017).

Data obtained in 1 monkey suggest that the effects of EM on categorization within the STS were location-specific, similarly to EM studies of face categorization (Afraz et al. 2006) and discrimination (Moeller et al. 2017). One factor likely contributing to STS-location specificity is the category selectivity of the STS region since only regions that show the necessary selectivity are expected to contribute to the behavioral performance (Rajalingham and DiCarlo 2019). Since EM outside ASB could be expected to result in signals related to the stimulus selectivity at these sites, the lack of EM effects at those STS sites seems to refute the above suggestion that effects of EM for nonbody categories in ASB result from EM-elicited body signals. One explanation of this apparent discrepancy is that such putative combinatory effects may depend on a yet undefined interaction between stimulus category and EM-induced signals (Moeller et al. 2017). Another possibility is that it is caused by a task-dependent readout of responses in different regions.

Switching the 2CC target locations in monkey MG resulted in a concomitant reversal of the EM-induced choice biases when sorted per category, indicating that target location and not its associated category was an important determinant of the EM effect. Particularly, EM resulted in frequent choices toward the target contralateral to the stimulated hemisphere. Also, the EM-induced choice biases in the nonbody 2CC tasks tended to avoid the ipsilateral hemifield. Furthermore, an EM-induced contralateral hemifield bias could explain the differences between the 2 monkeys regarding the EM effects in the 4CC task. The contralateral bias could be due to EM-induced body signals or phosphenes in the contralateral visual field. High current EM in the ventral temporal cortex in humans can induce percepts in the contralateral field (Puce et al. 1999; Schalk et al. 2017), and such percepts may bias choices for that field. However, EM and non-EM saccade traces overlapped strongly (Supplementary Fig. S4), providing additional evidence against the possibility that the shift in the psychometric curve could be simply due to EM-induced body signals or phosphenes. Putative contralateral bias signals must have occurred during the processing of the cue and not the target images since the monkeys were planning their saccades during the cue presentation. We infer this from the short saccadic reaction times (range of median reaction times, computed from target onset, at lowest SNR in 4CC task for the 4 categories in EM trials: MG: 40-45 ms; MB: 104–117 ms; also see Supplementary Fig. S3 for 2CC tasks), with negligible difference between EM and non-EM trials.

Other aspects of our data argue against a contralateral hemifield bias explanation of our EM effects. First, neither monkey showed an effect of EM, and thus a contralateral field bias, in the animals versus houses categorization (2CC, following the 4CC task). However, negative findings in EM studies are difficult to interpret. Second, switching of the animal and house targets in this 2CC task increased animal choices in both monkeys despite the opposite hemifield location of the targets. Third, ASB receptive fields show a contralateral bias (Kumar et al. 2017), similar to other regions in IT (Op de Beeck and Vogels 2000). Given the widespread contralateral receptive field bias within IT, it is unclear how a putative EM-induced hemifield bias can explain the absence of EM effects at locations outside ASB in MG. These arguments suggest that an EM-induced contralateral bias is merely one of the factors underlying the observed behavioral biases in the categorization task with EM of ASB.

No EM effects were observed for face or object choices in the 4CC task, but EM effects were present for faces versus objects categorizations in the subsequent 2CC task. These findings suggest that EM effects in the ASB depend on the task context in forced-choice categorization tasks. Moreover, after switching the target locations and retraining, the EM of ASB increased animal choices in the houses versus animals categorization for both monkeys, while EM did not affect performance for the same categorization before switching target location. This may have resulted from changes in readout because of the reversal training. Although changes in readout have been reported with perceptual training (Chowdhury and DeAngelis 2008; Liu and Pack 2017), this remains speculative here.

We started out testing the hypothesis that the body patch ASB contributes to animal categorization. We employed a categorization task and experimental procedures similar to that used for face categorization in IT (Afraz et al. 2006). Our initial results, an increase in animal choices when ASB is stimulated, parallel those of face categorization in a previous study (Afraz et al. 2006). However, our subsequent experiments showed that EM effects were not category-specific and suggested an unexpected and complex interplay of multiple factors underlying EM effects in IT. As in our 4CC and the subsequent 2CC tasks, Afraz et al. (2006) employed saccadic choice targets in opposite hemifields. Their EM effects could not be explained by a contralateral hemifield bias since the associated category differed with respect to the stimulated hemisphere between their monkey subjects. They, however, did not examine the effect of EM in face-selective clusters for other categorizations than faces versus objects and thus we do not know how face category-specific their EM effects were. Moeller et al. stimulated fMRI-defined face patches in monkeys and used nonface discriminations in addition to faces (Moeller et al. 2017). Like our ASB results, they observed effects of face patch EM on nonface discrimination, such as houses. Thus, it is possible that EM in face patches also will affect nonface categorizations and thus that behavioral effects of EM in face and body patches might be similar, at least regarding their category-specificity. On the other hand, a potential higher category-specificity of EM effects for face patches compared to body patch ASB may result from face patches being more category-selective for faces than body patches are for bodies (Popivanov et al. 2014; Kumar et al. 2017; Bao et al. 2020).

In general, our findings highlight the interpretive inadequacy of current models, which mostly rely only on image selectivity, of the behavioral effects evoked by EM in the IT cortex. Interactions between factors, such as stimulus selectivity of the stimulated site, target location, and task context, should be taken into consideration when employing EM (or other stimulation techniques such as optogenetics with depolarizing opsins) as a tool to interrogate the contribution of signals in sensory areas to perceptual tasks.

## Supplementary Material

Supplementary_Figure_and_Table_revision_final_tgac010

## Data Availability

Data can be obtained from the authors upon reasonable request.
