# Supplementary material for: It is not just the category: behavioral effects of fMRI-guided electrical microstimulation result from a complex interplay of factors
Source: Cereb Cortex Commun. 2022 Feb 26;3(1):tgac010. doi: 10.1093/texcom/tgac010 (PMC8935663; doi:10.1093/texcom/tgac010)
Supplement: Supplementary_Figure_and_Table_revision_final_tgac010 [file supplementary_figure_and_table_revision_final_tgac010.docx]

**It is not just the category: behavioral effects of fMRI-guided electrical microstimulation result from a complex interplay of factors**

S Kumar, E Mergan, R Vogels.

**Supplementary Figures and Table**


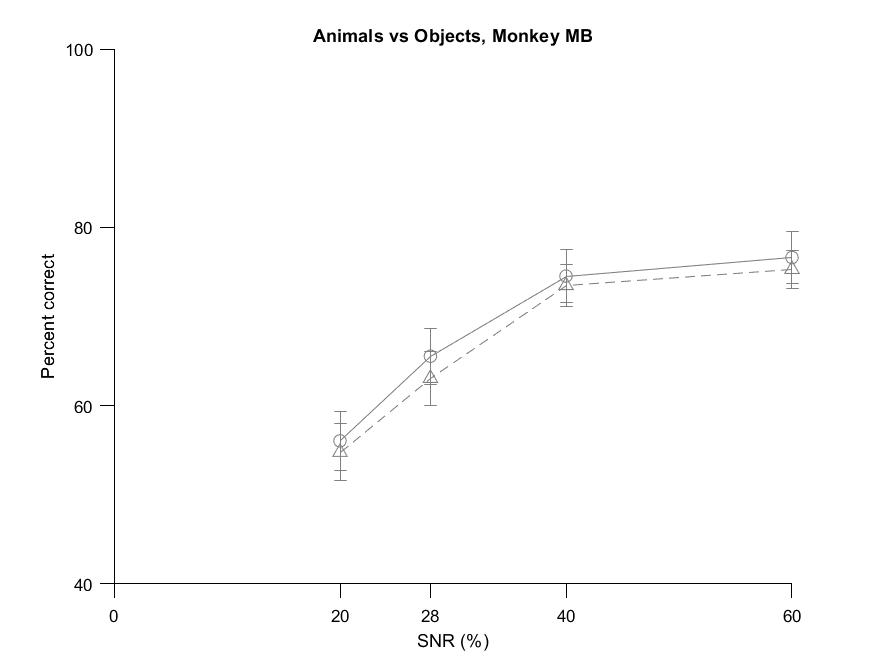


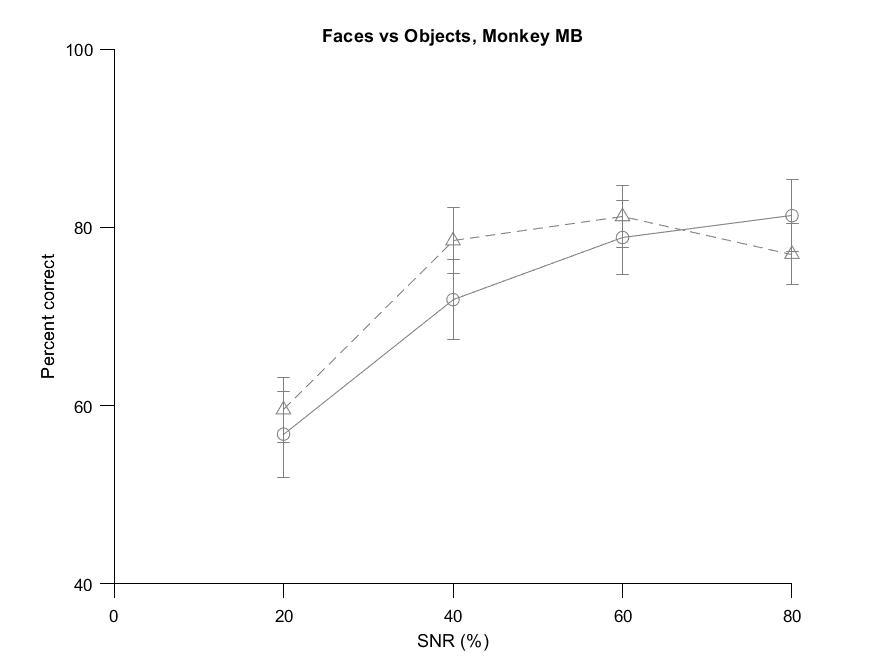


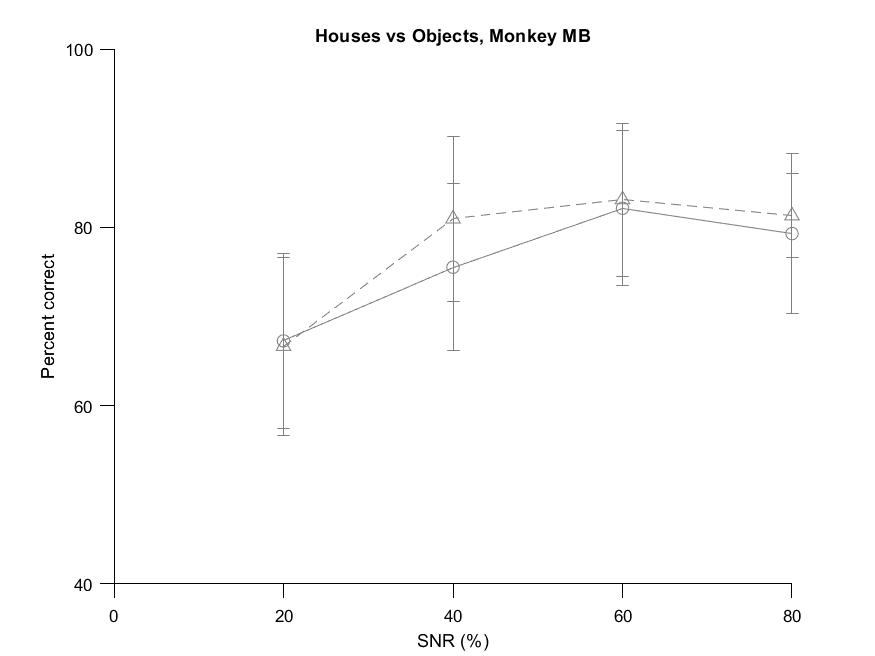


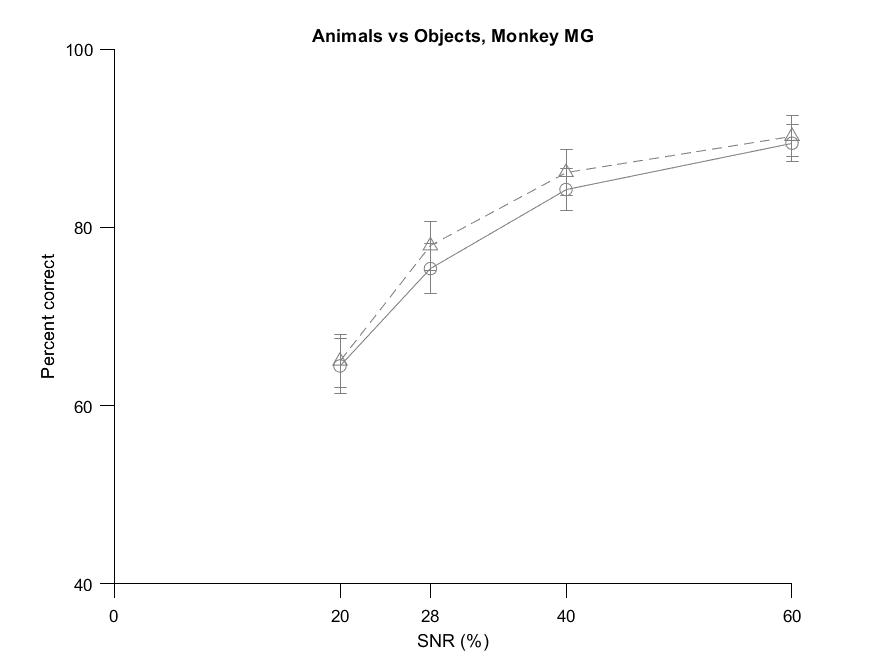


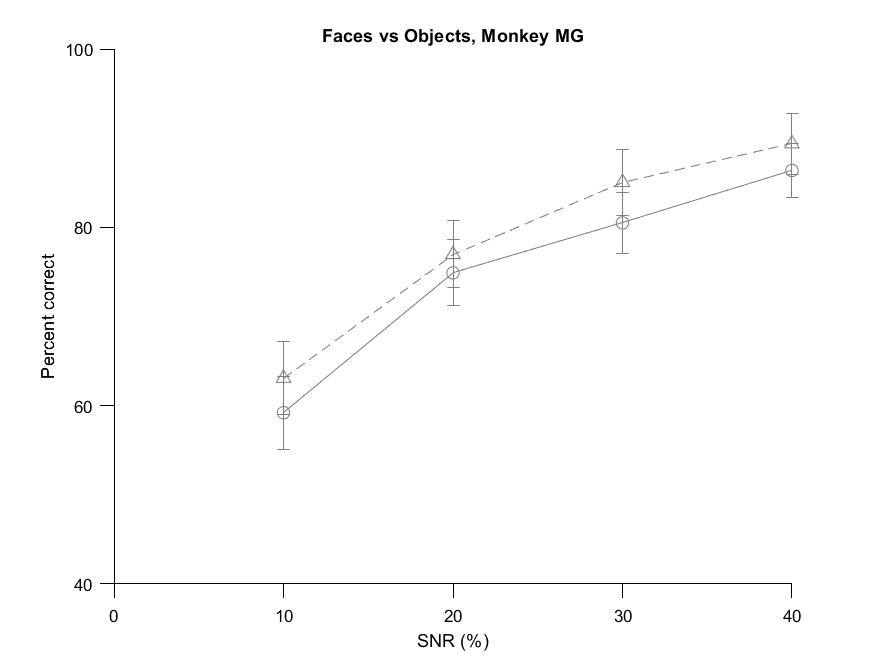


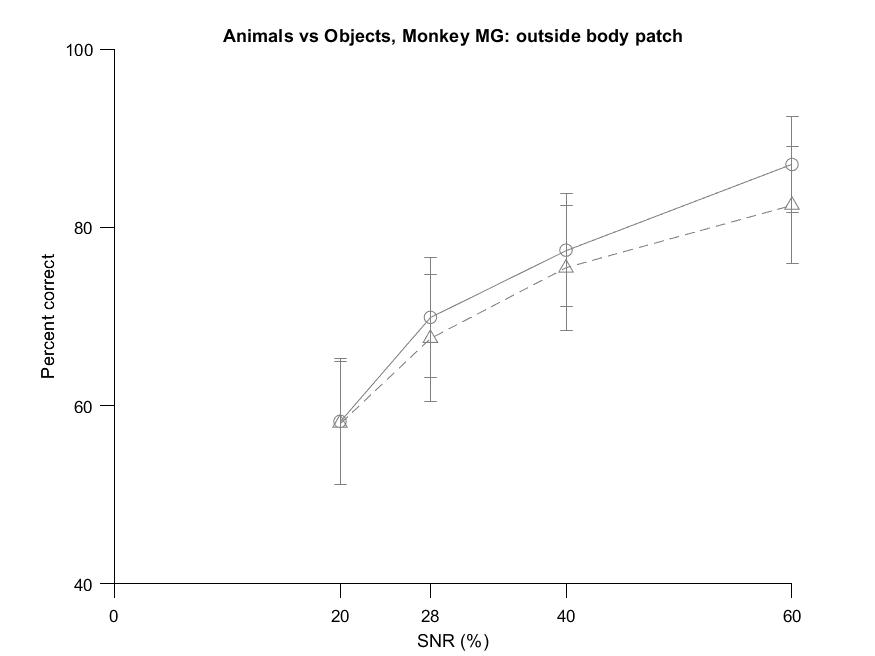


**Supplementary Figure S1. Effect of EM on percent correct responses in 2CC EM experiments.** Overall performances (% correct responses) as a function of SNR (%) for the different 2CC tasks of monkey MB and monkey MG. The full lines (and circles) and dotted lines (and triangles) represent the performance in the non-stimulated and EM trials, respectively. Error bars indicate 95% confidence intervals.

**
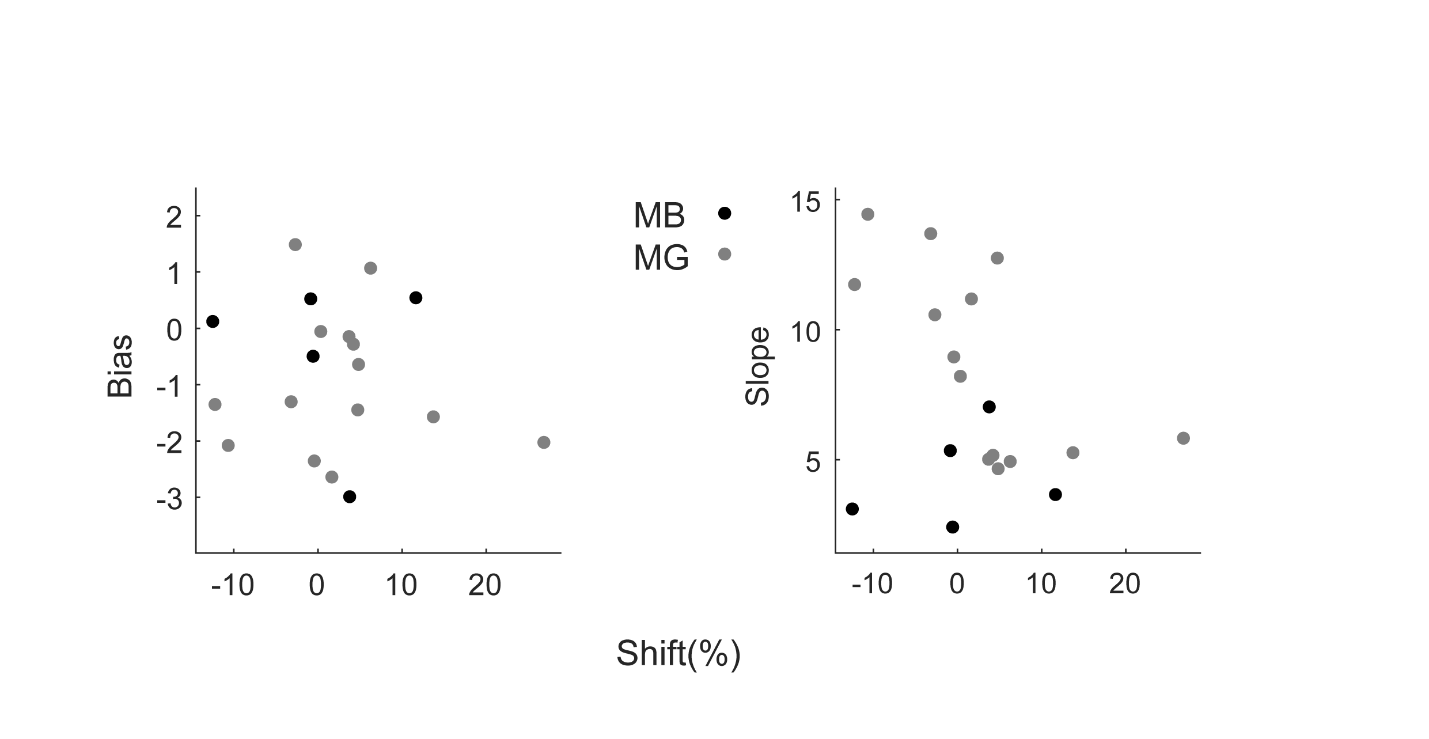
**

**Supplementary Figure S2. The response bias and slope of the psychometric functions plotted against the EM-induced shift for the 2CC EM experiments.** Effects of EM (50 µA) as parametrized by the shift (𝜆/𝛽; see equation 1 in the Methods) values are unrelated to the non-EM response bias (𝛼) (left panel; Pearson correlation coefficient (r) = -0.14, p = 0.54) and to the slope (𝛽) (right panel; r = -0.25, p = 0.28) of the fitted psychometric function for each tested response target configuration in both the monkeys. Data of different monkeys are indicated separately.


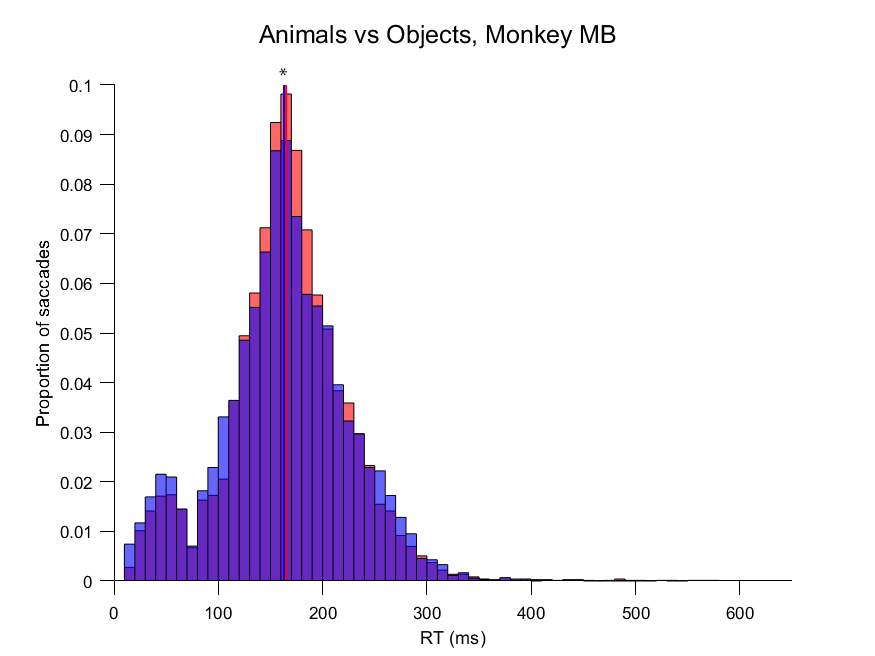


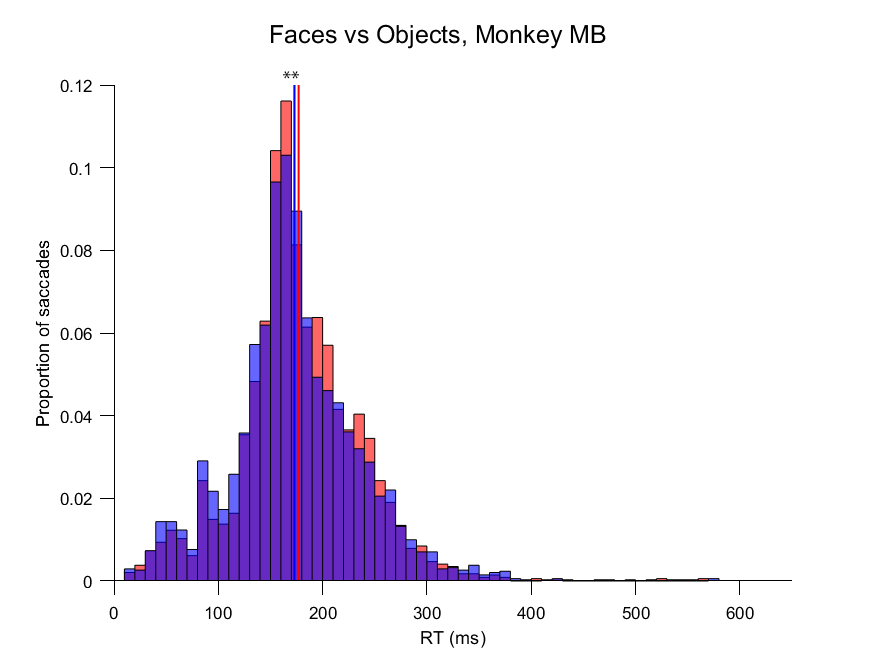


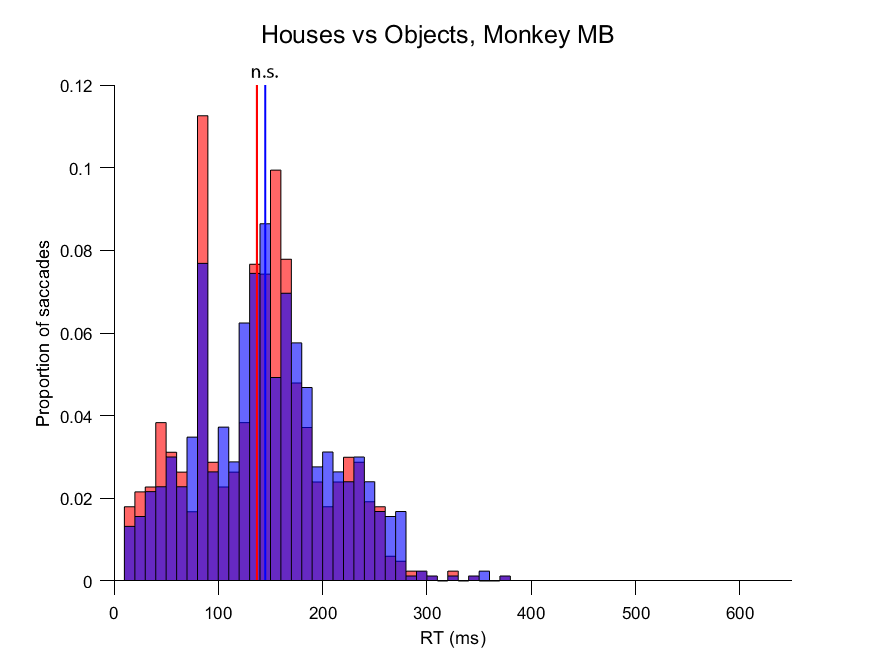


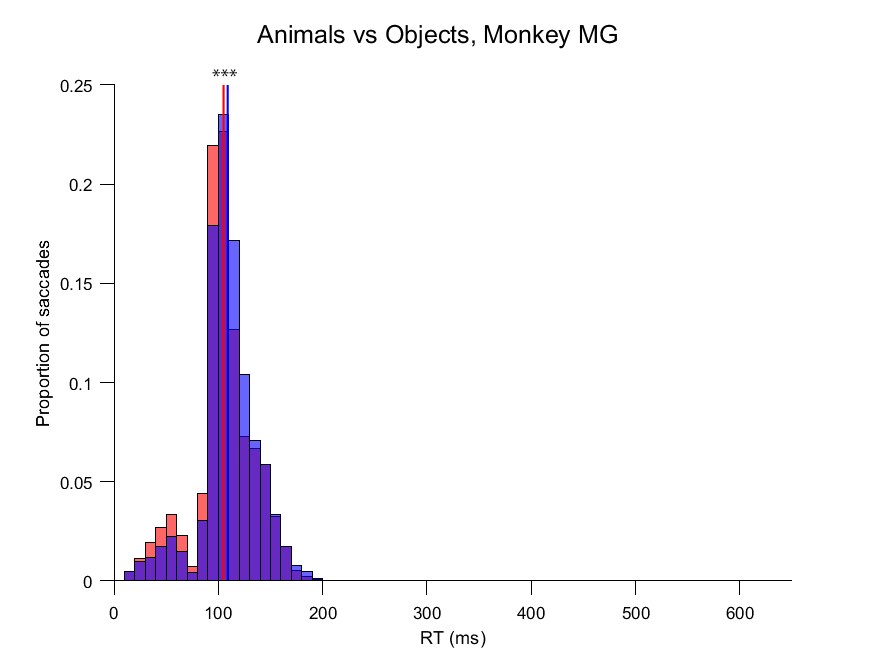


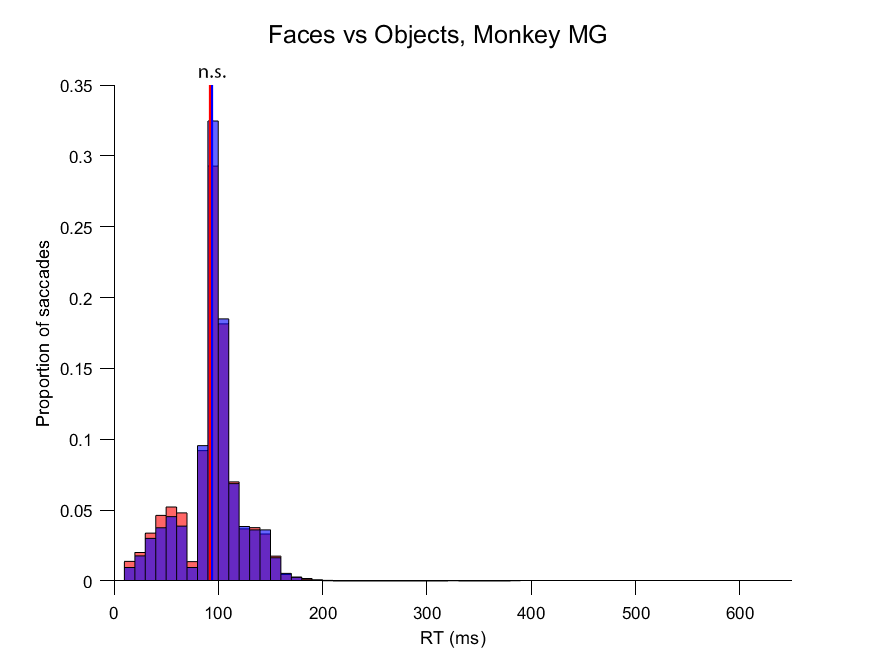


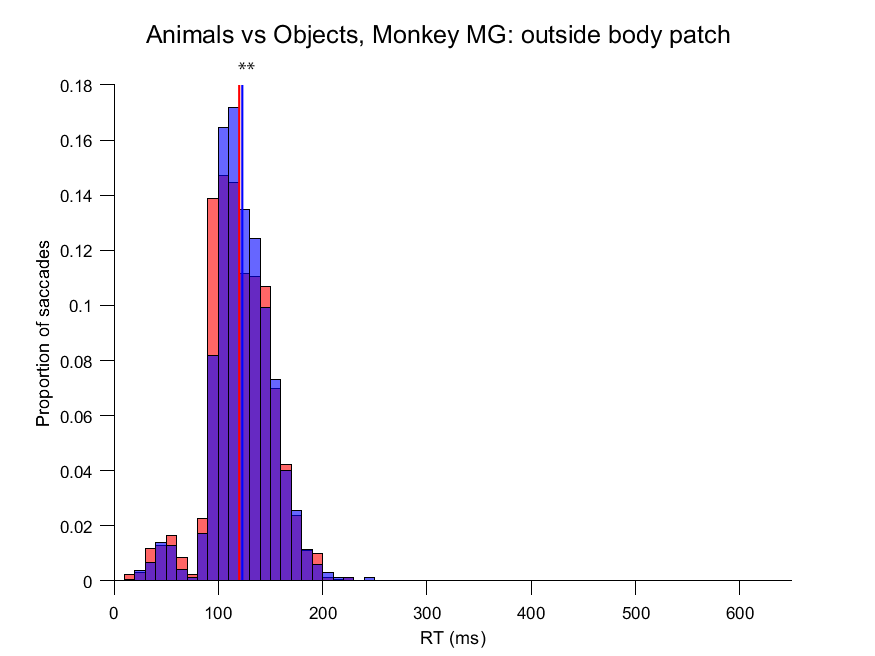


**Supplementary Figure S3. Distribution of the reaction times (RT) in 2CC EM experiments.** Reaction times distribution for microstimulation (red) and non-microstimulation (blue) conditions. The vertical lines represent the mean reaction times for microstimulation (red) and non-microstimulation (blue) trials. These data were pooled from all the valid trials across all SNR levels for each monkey and test. The significance of the difference between the two distributions was determined by a two-sided Mann-Whitney U-test. Significance is indicated by different symbols (*** represents a p-value less than 0.001, ** represents a p-value less than 0.01 and greater than 0.001, * represents a p-value less than 0.025 and greater than 0.1. and n.s. represents a p-value greater than 0.025).


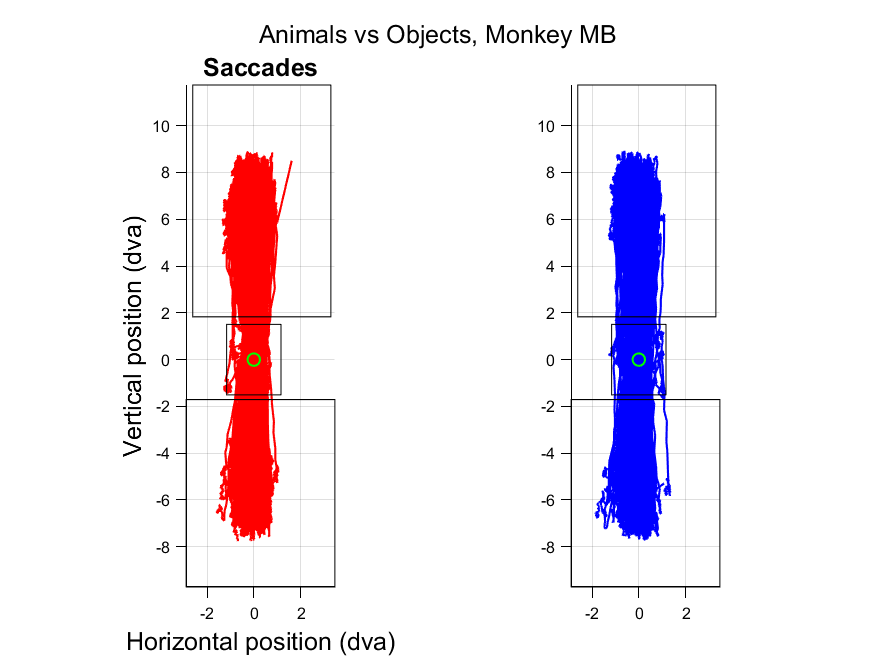


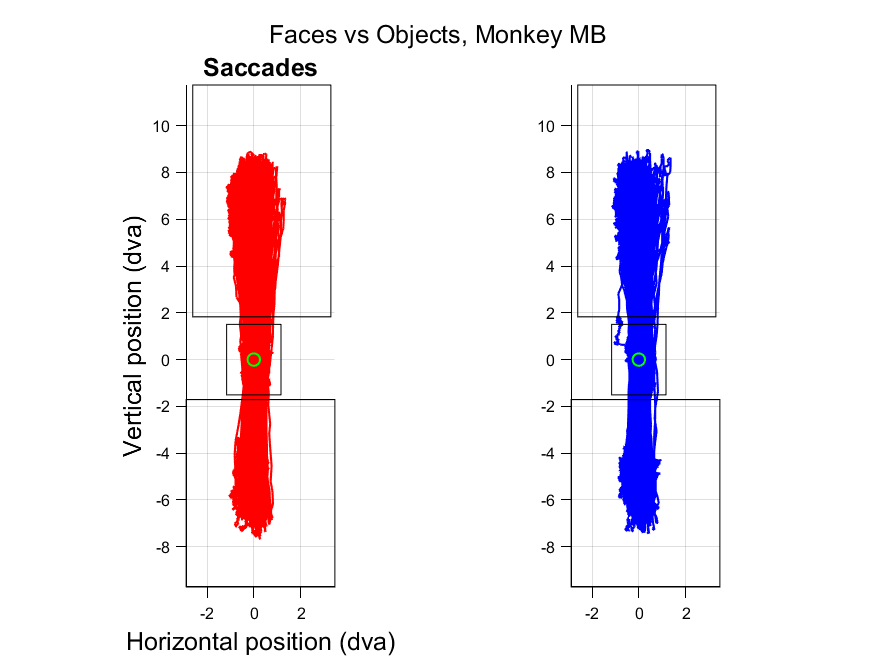


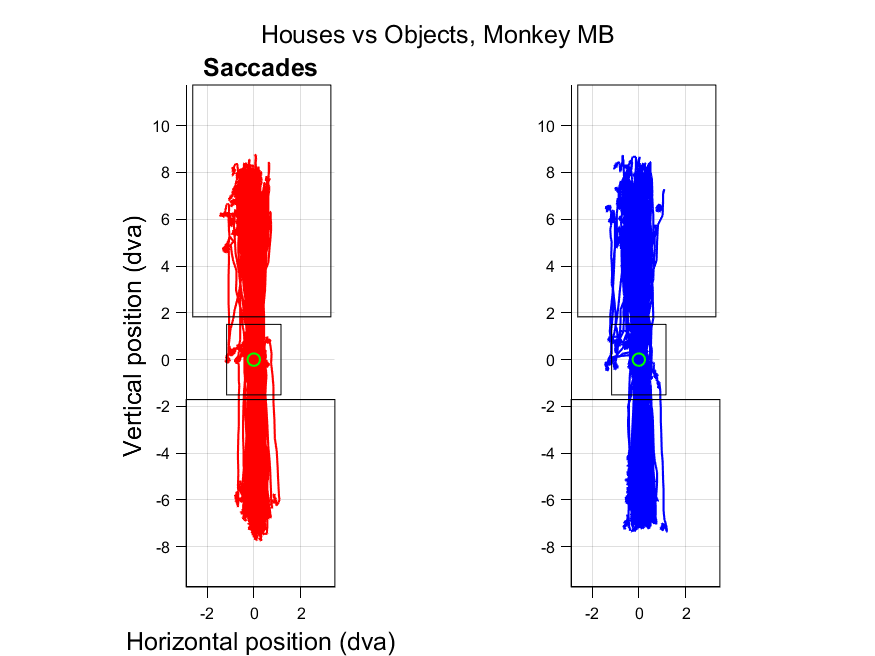


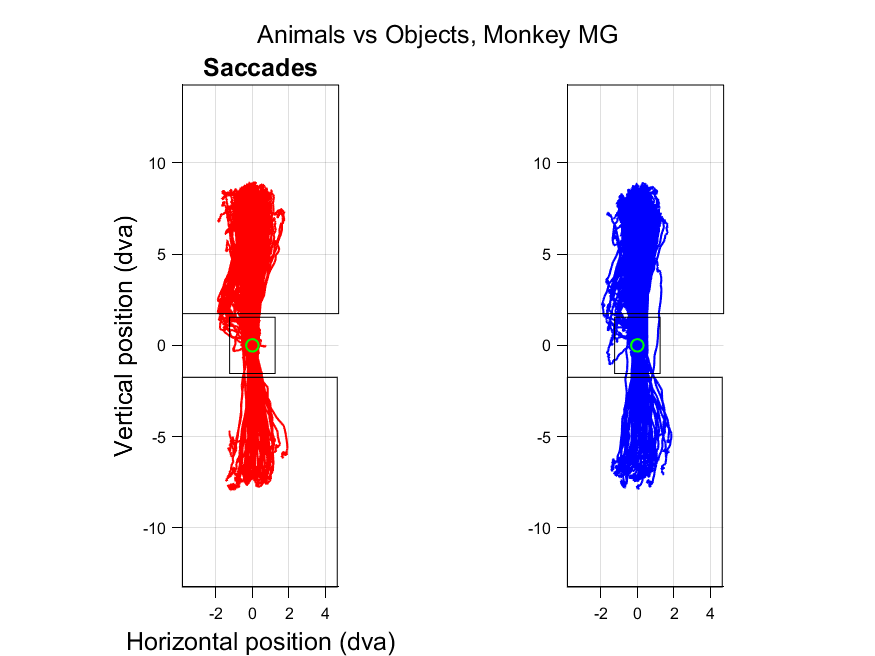


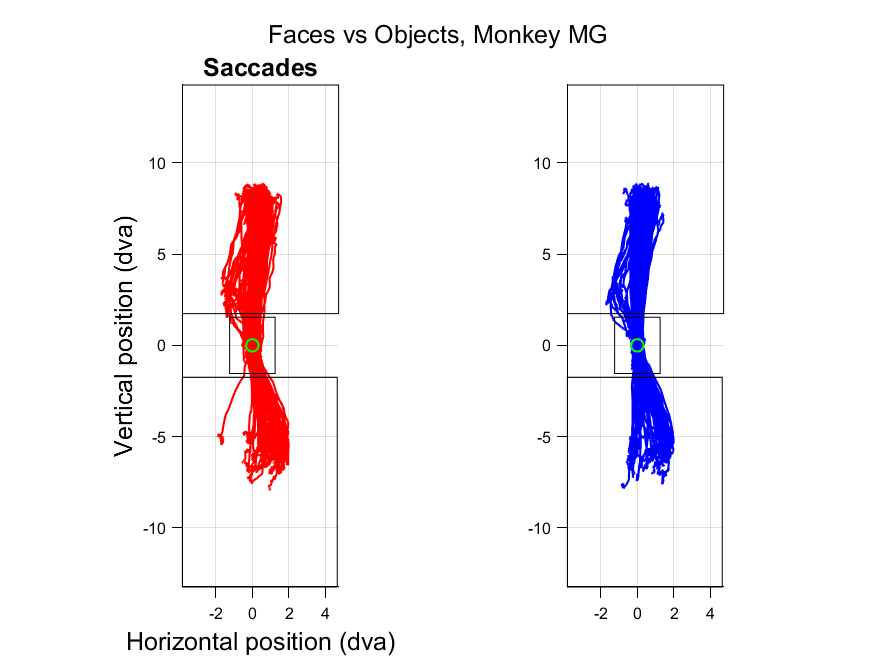


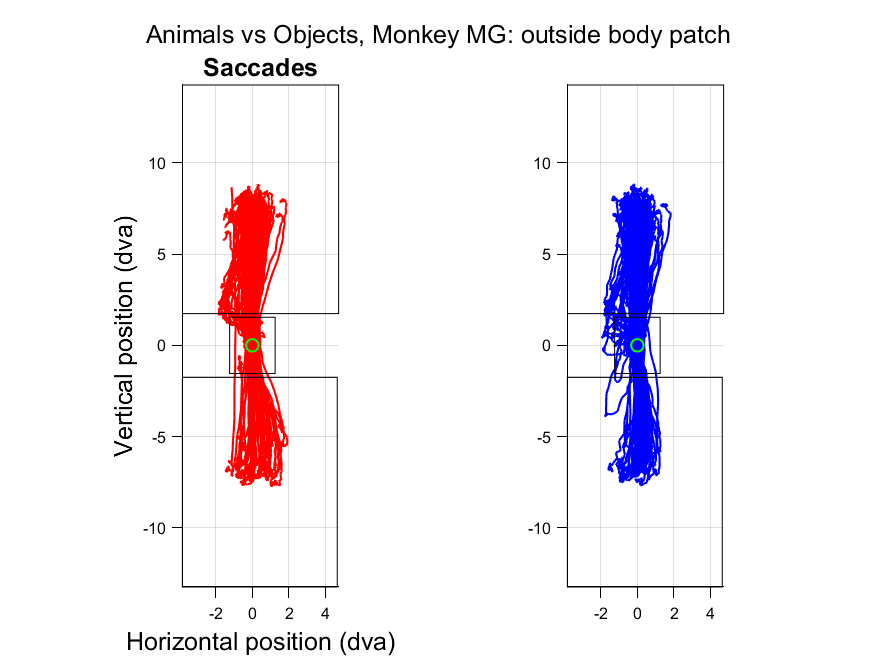


**Supplementary Figure S4. Saccade traces in 2CC EM experiments.** Eye movements for microstimulation (red) and non-microstimulation (blue) conditions. The circle (green) denotes the fixation point location. These data were pooled from all the valid trials across all SNR levels for each monkey and test. The middle window represents the mean fixation window, while the top and bottom windows represent the mean target windows. Note that the target windows have a much larger size than the 4^o^ visual angle target stimuli and thus can capture potential phosphene-induced saccades.

| Monkey | Task | Proportion of the EM trials | p-value (two -sided Binomial test) |
| --- | --- | --- | --- |
| MB | Animals vs Objects | 0.503 | 0.91 |
|  | Faces vs Objects | 0.511 | 0.86 |
|  | Houses vs Objects | 0.498 | 0.96 |
| MG | Animals vs Objects | 0.492 | 0.86 |
|  | Faces vs Objects | 0.495 | 0.91 |
|  | Animals vs Objects ( outside body patch ) | 0.481 | 0.47 |

**Supplementary Table S1. Proportion of the premature saccades in 2CC EM experiments.** We investigated whether premature saccades were more common in EM than in non-EM conditions. Saccades that occurred before 200 ms following stimulus onset were considered premature saccades. Using a two-sided Binomial test, we compared the proportions of premature saccades with EM (number of trials with EM divided by the total number of trials (EM + non-EM)) to the expected probability of 0.50 (null hypothesis). All p-values were corrected for multiple comparisons.

| MB | Task | Current | Sessions | Trials | Locations | Comments |
| --- | --- | --- | --- | --- | --- | --- |
|  | 2CC | | | | | |
|  | Animals vs. Object | 150 µA | 15 | 13559 | A0M0 | Inside body patch |
|  | Face vs. Object | 150 µA | 5 | 4252 | A0M0 |  |
|  | Animals vs. Object | 150 µA | 1 | 1147 | A0M0 |  |
|  | Face vs. Object | 150 µA | 4 | 2600 | A0M0 |  |
|  | House vs. Object | 150 µA | 3 | 1688 | A0M0 |  |
|  | **4CC** | | | | | |
|  | 4CC | 50 µA | 6 | 2526 | A0L2 | Inside body patch |
|  | **2CC** | | | | | |
|  | Animals vs. Object | 50 µA | 3 | 942 | A0L2 | Inside body patch |
|  | House vs. Object | 50 µA | 2 | 576 | A0L2 |  |
|  | Animals vs. Object | 50 µA | 1 | 291 | A0L2 |  |
|  | House vs. Object | 50 µA | 1 | 406 | A0L2 |  |
|  | Animals vs. House | 50 µA | 5 | 2019 | A0L2 |  |
|  | House vs. Object | 50 µA | 2 | 812 | A0L2 |  |
|  | Animals vs. House Reversed | 50 µA | 2 | 2760 | A0L2 |  |
|  |  |  |  |  |  |  |
| **MG** | **Task** | **Current** | **Sessions** | **Trials** | **Locations** | **Comments** |
|  | **2CC** | | | | | |
|  | Animals vs. Object | 150 µA | 4 | 3294 | A0M4 | Inside body patch |
|  | Animals vs. Object | 150 µA | 15 | 16124 | A1M4 |  |
|  | Face vs. Object | 150 µA | 9 | 9340 | A1M4 |  |
|  | **4CC** | | | | | |
|  | 4CC | 150 µA | 2 | 3909 | A1M5 | Inside body patch |
|  | 4CC | 50 µA | 5 | 9269 | A1M5 |  |
|  | 4CC | 50 µA | 4 | 6405 | P2M1 | Outside body patch |
|  | 4CC | 50 µA | 2 | 3651 | A1M5 | Inside body patch |
|  | 4CC | 50 µA | 2 | 3495 | P2M1 | Outside body patch |
|  | **2CC** | | | | | |
|  | Animals vs. Object | 50 µA | 1 | 1360 | A1M5 | Inside body patch |
|  | House vs. Object | 50 µA | 1 | 1396 | A1M5 |  |
|  | Animals vs. Face | 50 µA | 1 | 1091 | A1M5 |  |
|  | Face vs. House | 50 µA | 2 | 1882 | A1M5 |  |
|  | Face vs. Object | 50 µA | 1 | 1318 | A1M5 |  |
|  | Animals vs. House | 50 µA | 2 | 2564 | A1M5 |  |
|  | Animals vs. Object | 50 µA | 1 | 1392 | A1M5 |  |
|  | Animals vs. Object Reversed | 50 µA | 1 | 1426 | A1M5 |  |
|  | Animals vs. House Reversed | 50 µA | 1 | 2311 | A1M5 |  |
|  | Animals vs. Object Reversed | 50 µA | 1 | 1042 | A1M5 |  |
|  | Animals vs. House Reversed | 50 µA | 1 | 2648 | A1M5 |  |
|  | Animals vs. Object Reversed | 50 µA | 1 | 1392 | A1M5 |  |
|  | Animals vs. Object Reversed | 50 µA | 5 | 8901 | A0M4 | Outside body patch |
|  | Animals vs. Object Reversed | 50 µA | 4 | 8902 | P1M3 |  |
|  | Animals vs. Object Reversed | 50 µA | 4 | 3406 | P2M2 |  |
|  | Animals vs. Object Reversed | 50 µA | 3 | 4908 | P3M1 |  |
|  | Animals vs. Object Reversed | 50 µA | 2 | 5273 | A1M5 | Inside body patch |

**Supplementary Table S2. Chronological order and experimental details for MB and MG.** For each experiment, details are enumerated in chronological order. During the experimental sessions, we adhered to the following rule: for every negative finding, i.e., absence of any behavioral effect due to microstimulation, we repeated previous reliable positive findings, i.e., significant behavioral effect elicited by the microstimulation.
